# Supplementary material for: Selectivity descriptors for the direct hydrogenation of CO2 to hydrocarbons during zeolite-mediated bifunctional catalysis
Source: Nat Commun. 2021 Oct 8;12:5914. doi: 10.1038/s41467-021-26090-5 (PMC8501036; doi:10.1038/s41467-021-26090-5)
Supplement: Supplementary file 1 — Supplementary Information [file 41467_2021_26090_MOESM1_ESM.pdf]

Supplementary Information

Selectivity descriptors for the direct hydrogenation of CO_2 to hydrocarbons during zeolite-mediated bifunctional catalysis

Ramirez and Gong et al.

Table of contents

Supplementary Methods 3
Supplementary Discussion 10
Supplementary Figures 24
Supplementary Tables 55
Supplementary References 59

Supplementary Methods

Chemicals. Iron oxide (Fe_2O_3, Aldrich), potassium superoxide (KO_2, Aldrich), ZSM-5, (ACS Materials), SAPO-34 (ACS Materials), MOR (Alfa Aesar), FER (Alfa Aesar), HY (Zeolyst), BETA (Zeolyst), and ZSM-22 (ACS Materials) were used as received. ZSM-58 was synthesized according to the earlier report from Kumita et al.[1] The Isotopic Gas Mixtures (25% $^{13}CO_2$ in Hydrogen and 25% ^{13}CO in Helium, 100 bar lecture bottle) were provided by CK Isotopes Limited.

Catalyst preparation. The Fe_2O_3@KO_2 catalyst was obtained by the mortar mixing of Fe_2O_3 and KO_2, keeping a molar ratio of Fe/K=2. The resultant mixture was heated up to 100 ºC for 12 h prior to the catalytic measurements. All the zeolites were dried at 120 ºC for 12 h and calcined at 550 ºC for 2 hours before the catalytic testing.[2–5]

Catalytic testing. Catalytic tests were executed in a 16 channel Flowrence® from Avantium. 50 mg of the stand-alone Fe_2O_3@KO_2 catalyst and 100 mg of the composite catalyst with Fe_2O_3@KO_2/Zeolite with mass ratio 1/1 in a dual bed configuration was typically used. The mixed feed had 132 ml/min of 25 vol% of $^{13}CO_2$ and 75 vol% of H_2. In addition, 8 ml/min of He was mixed with the feed as an internal standard. We aimed to have 10000 $mL \cdot g^{-1} \cdot h^{-1}$ per channel in the stand-alone catalyst and 5000 $mL \cdot g^{-1} \cdot h^{-1}$ in the composite catalyst. One of the 16th channels was always used without any catalyst as a 'blank'. The reaction temperature was typically set at 375°C. Prior to feeding the reaction mixture, all samples were pretreated in-situ with a pure H_2 atmosphere for 4 hours at 350°C. The tubes were then pressurized to 30 bar using a membrane-based pressure controller.

GC is an Agilent 7890B with two sample loops. After flushing the loops for 24 min, the content is injected. One sample loop goes to the TCD channel with 2 Haysep pre-column and MS5A, where He, H_2, CH_4, and CO are separated. Gases with longer retention times than CO_2 on the Haysep column (Column 4 Haysep Q 0.5 m G3591-80023) are back-flushed. Further separation of permanent gases is done on another Haysep column (Column 5 Haysep Q 6 Ft G3591-80013) to separate CO_2 before going to MS5A. Another sample loop goes to an Innowax pre-column (5m, 0.20mm OD, 0.4 µm film), the first 0.5 min of the method, the gases coming from the pre-column are sent to Gaspro column (Gaspro 30M, 0.32 mm OD) followed by FID. After 0.5 min, the valve is switched, and gases are sent to the Innowax column (45 m, 0.2 mm OD, 0.4 µm) followed by FID. Gaspro column separates C_1-C_8, paraffins, and olefins. Innowax separates larger paraffins and olefins (>C_9) and aromatics BTX (Benzene, Toluene, Xylene: BTX) and C_9+ aromatics. Conversion (X, %), space-time yields (STY, $mmol \cdot gcat^{-1} \cdot h^{-1}$), hydrocarbon selectivities (S_{Cn}, %) and CO selectivity (S_{CO}, %) are defined as follow:

$$X_{CO_2} = \left(1 - \frac{C_{He,blk} \cdot C_{CO_2,R}}{C_{He,R} \cdot C_{CO_2,blk}}\right) \cdot 100$$

$$S_{Cn} = \frac{n \cdot C_{Cn,R}}{\sum n \cdot C_{Cn,R}} \cdot 100$$

$$S_{CO} = \frac{\frac{C_{CO,R}}{C_{He,R}}}{\left(\frac{C_{CO_2,blk}}{C_{He,blk} \cdot} - \frac{C_{CO_2,R}}{C_{He,R}}\right)} \cdot 100$$

where $C_{He,blk}$, $C_{He,R}$, $C_{CO2,blk}$, $C_{CO2,R}$ are the concentrations determined by GC analysis of He in the blank, He in the reactor effluent, CO_2 in the blank, and CO_2 in the reactor effluent, respectively, and $C_{Cn,R}$ is the concentration the reactor effluent determined by GC analysis of a product with n carbon atoms. The error in carbon balance was better than 2.5% in all cases.

The control experiments with isotopic ^{13}CO were carried out by feeding to the Flowrence® , reactor a ^{13}C labeled CO mixture (25% ^{13}CO in Helium) plus ethylene and Hydrogen. In particular, we aimed for 5 mL/min of the 25% ^{13}CO in Helium mixture, 2.5 mL/min of H_2 and 0.5 mL/min of ethylene per reactor. 50 mg of the stand-alone zeolites were used as catalyst. For the control experiments without isotopic ^{13}CO (i.e., only using ethylene in the reactant feed), the other reaction conditions remained similar.

Catalyst characterization. Nitrogen adsorption and desorption isotherms were recorded on a Micromeritics ASAP2420 at 77 K. Samples were previously evacuated at 350 °C and 90 °C for 8 and 2 hours, respectively. The temperature-programmed desorption (TPD) experiments were carried out in a AMI-200ip Catalyst Characterization System (Altamira) equipped with TCD. The zeolite samples were first heated in argon flow at 100 °C and 550 °C sequentially, followed by cooling to 120 °C. Afterward, the zeolites were statured in ammonia and the temperature of the samples was increased to 150 °C to remove physisorbed NH_3 under Ar flow. Then, the temperature was increased to 750 °C with a rate of 10 °C/min. Raman spectra were recorded using a confocal Raman microscope WITec Apyron equipped with 532 and 633 nm laser lines. The oil immersion objective (Zeiss Achroplan/N-Achroplan Oil 100x / NA 1.25) and immersion oil (Immersol 518 F, Carl Zeiss) were applied in all acquisitions. Raman spectra from different locations were collected for each sample. Powder X-ray diffraction measurements for air sensitive catalyst were conducted using a Bruker D8 Venture single crystal diffractometer equipped with a PHOTON II area detector and an IµS microfocus source (set to 50 kV, 1 mA) providing an Mo Kα radiation (λK1=0.70930 Å, λK2=0.71359 Å). The detector was positioned at a swing angle θ=0° and a sample-to-detector distance of 40 mm to collect an extended 2θ range of 2.7–107.2°. Capillaries of 1 mm diameter were filled with powders using standard procedure to handle air sensitive

compound (e. g. the spent catalyst), then air-sealed and finally mounted on the goniometer for transmission measurement. A single frame per sample was recorded with an exposure time of 10 min using a phi-360 scan. Integration over the 10-60° 2θ range was performed by DIFFRAC.EVA software from Bruker.

Computational methodology. The stability of several intermediates formed, as proposed in Figure 8 in the main text, was based on previous studies.[6–8] The analysis has been performed using the following methodology.

Density Functional Theory calculations: Periodic DFT calculations were conducted using Vienna ab initio Simulation Package (VASP),[9,10] Perdew-Burke-Ernzerhof functional[11] with Grimme's dispersion correction (PBE-D3),[12] and a plane-wave basis set of the projector-augmented-wave (PAW) method.[13] An energy cut-off of 600 eV was used for the expansion of the wave function in the plane wave basis set. A $(1 \times 1 \times 1)$ γ-centered k-point mesh was employed to sample the first Brillouin zone and Gaussian smearing with a width of 0.5 eV. All atoms were relaxed until electronic energies varied by $< 1 \times 10^{-5}$ eV, and the forces on all atoms were < 0.01 eV Å^{-1}.

Zeolite models: For each zeolite model, the strongest and accessible adsorption sites of CO and other oxygenates, were investigated to determine the dominant interactions that can occur inside the zeolite pores, together with the two lowest adsorption sites to have a broader understanding of the range of interactions. A single unit cell of 96 T atoms was used to model the H-ZSM-5 zeolite, with a Brønsted acid site being created by substituting the T12[14,15] site with Al and adding a charge-compensating proton. In the case of H-ZSM-22, a supercell of 4 unit cells $(1 \times 1 \times 4)$ was employed in order to prevent any errors from sorbate mirror images, as previously conducted,[16,17] resulting in a model having 96 T atoms, with a Brønsted acid site being created by substituting the T1[16] site

with Al and adding a charge-compensating proton. H-FER with a double unit cell consisting of 72 silicon and 144 oxygen atoms, with the Al substituted in T1,[14,18] while for H-MOR, a 1×1×2 super cell consisting of 96 T atoms has been used, with Al substituted in the T1 site.[19,20] In order to estimate the range of adsorption energies that can be achieved on the zeolite acid sites, the lowest adsorption sites from the highest and lowest adsorption strength zeolites were further included in our analysis. Thus, from H-ZSM-5 and H-FER, T6[15] and T3[18], respectively, Al substituted Brønsted sites were investigated as well.

Magic angle spinning (MAS) solid-state nuclear magnetic resonance (NMR) measurements. All ^{1}H and ^{13}C related (both 1D and 2D) magic angle spinning (MAS) solid-state nuclear magnetic resonance (ssNMR) spectroscopic experiments were performed on Bruker AVANCE III spectrometers operating at 400 MHz frequency for ^{1}H using a conventional double-resonance 3.2 mm CPMAS HX probe (CP: Cross-polarization) (Supplementary Table 4). NMR chemical shifts are reported with respect to the external reference adamantane. Herein, samples were prepared using fully enriched $^{13}CO_2$ or ^{13}CO at our typical experimental conditions (30 bar, 375ºC, H_2:$^{13}CO_2$/^{13}CO=3, and 10000 mL·g^{-1}·h^{-1}) to increase the ssNMR sensitivity, unless specified otherwise in the figure captions. All NMR measurements were performed at room temperature (298 K) and MAS frequency of 16 or 20 kHz (unless specified otherwise in the figure captions). Note that effective sample temperatures can be 5-10 degrees higher due to the frictional heating. The 1D ^{1}H-^{13}C cross-polarization (CP)[21] spectrum was recorded using a 4 s recycle delay, an 12 ms acquisition time, and accumulation of 6k scans. The 1D direct excitation (DE) spectrum was recorded using a 4 s recycle delay, an 16 ms acquisition time, and an accumulation of 1 or 2k scans. The 1D insensitive nuclei enhanced by polarization transfer (INEPT) spectrum[22] was recorded using a 2 s recycle delay, an 20 ms acquisition time, and accumulation of 10k scans. 1D

CP, DE, and INEPT spectra were processed using 100 Hz, 120 Hz, and 200 Hz line-broadening, respectively. ^{1}H and ^{13}C pulses were applied with a field strength of 70 and 50 kHz, respectively. 2D ^{13}C-^{13}C spectra were recorded using a 2 s recycle delay, 10 ms (F2) and 1.3 ms (F1) acquisition time and an accumulation of 256 scans (both CP and DE). ^{13}C-^{13}C mixing was achieved through proton driven spin-diffusion (PDSD) using Phase-alternated-recoupling-irradiation-schemes (PARIS) for 120 ms (CP) or 200 ms (DE) mixing.[23] 70 kHz SPINAL64 ^{1}H decoupling was applied during both direct and indirect dimensions.[24] To probe mobile molecules, 2D ^{13}C-^{1}H HSQC (Heteronuclear Single Quantum Coherence) spectrum[25] was recorded using 145 Hz for J in the INEPT. Herein, the 2D ^{13}C-^{1}H HSQC spectrum was recorded with a recycle delay of 2 s, 21 ms (^{1}H, F2) and 0.5 ms (^{13}C, F1 in ZSM-5)/0.1 ms (^{13}C, F1 in FER and MOR)/0.5 ms (^{13}C, F1 in ZSM-22) acquisition time and 128 scans. For 2D ^{1}H-^{13}C INEPT-based HETeronuclear CORrelation (HETCOR) to probe mobile molecules, 1.5 s recycle delay, 17 ms (^{13}C, F2) and 4 ms (^{1}H, F1) acquisition time and an accumulation of 512 scans (in ZSM-5 and ZSM-22)/640 scans (in FER)/2048 scans (in MOR) were used. To probe rigid molecules, dipolar based ^{13}C-^{1}H HETCOR correlation spectra were obtained using a fixed 0.5 ms contact time for both ^{1}H-^{13}C CP (p15) period and ^{13}C-^{1}H CP (p16) period. Background signals from catalyst protons were suppressed by a 8 kHz MISSISSIPPI block (τ = 5 ms, N = 2) prior to the last CP.[26] ^{13}C PISSARRO (Phase-Inverted Supercycled Sequence for Attenuation of Rotary Resonance) decoupling was applied during acquisition.[27] The recycle delay was 2 s, acquisition times 10 ms (F2, ^{1}H) and 2 ms (F1, ^{13}C) ms and number of scans 64 (128 for FER only). For the NMR measurements related to the ^{13}CO-based control experiments, 64 scans were accumulated for dipolar based ^{13}C-^{1}H correlation-based CP HETCOR spectra for all zeolites, except 128 scans for ZSM-5, while other parameters were similar to the previous experiments. Similarly, for the ^{13}CO-based control experiment on

FER sample, 2D ^{13}C-^{13}C spectra (both CP and DE) were recorded using a 2 s recycle delay, 10 ms (F2) and 1 ms (F1) acquisition time and an accumulation of 320 scans (for DE) and 512 scans (for CP). Here also, ^{13}C-^{13}C mixing was achieved through proton driven spin-diffusion (PDSD) using Phase-alternated-recoupling-irradiation-schemes (PARIS) for 120 ms (CP) or 200 ms (DE) mixing. For the control ssNMR experiments related to the verification of carbonyl peaks, organic carbonyls were physisorbed on zeolite within the rotor. In the case of potassium-based salts, these are directly transferred into the rotor. Then, ^{13}C DE spectrum was recorded using a 10 s recycle delay and an 16 ms acquisition time, unless specified otherwise. ^{27}Al MAS ssNMR experiments were carried out on a 900 MHz Bruker AVANCE IV 21.1T spectrometers quipped with 3.2 mm CPMAS probes, where chemical shifts were externally referenced to $Al(NO_3)_3$. For ^{27}Al MAS ssNMR experiments, 1 s recycle delay and 4096 scans were applied for both fresh and post-reacted zeolites. In all cases, other relevant acquisition/experimental parameters were described in figure captions. All NMR spectra were processed and analyzed using Bruker TopSpin 4.0.

Supplementary Discussion

Iron-potassium phase characterization. For the characterization of standalone Fe_2O_3@KO_2 catalyst, we refer to our previous publication[2] where we have performed an in-depth characterization of both fresh and spent Fe_2O_3@KO_2 catalysts, using multimodal and complementary techniques, including X-ray diffraction, High-angle annular dark-field imaging-scanning transmission electron microscopy (HAADF-STEM), High energy resolution fluorescence detected X-ray absorption near edge structure (HERFD-XANES), CO_2 temperature-programmed oxidation, and X-ray photoelectron spectroscopy. Our previous study on the characterization of the metallic-phase revealed the existence of different forms of Fe and K-phases only; no organic carbonylated species were neither identified nor indicated on the metallic phase, which is exclusively detected on the zeolite phase only in the current study. To provide further experimental supports, in this work, we have performed Raman (micro)spectroscopy and air-protected capillary single-crystal X-ray diffraction studies on both fresh and spent Fe_2O_3@KO_2 catalysts (Supplementary Fig. 18-20).

Since our Fe_2O_3@K_2CO_3 catalyst was prepared by mortar mixing of Fe_2O_3 (Aldrich) and KO_2 (Aldrich), a uniform homogeneous mixture could not be expected. To gain an idea on this matter, Raman spectra were collected on different spots of the catalyst surface (Supplementary Fig. 18a). While the carbonate phase (K_2CO_3) is more dominant on some spots of fresh catalysts,

different iron-oxide phases were identified too. In line with our previous spectroscopic analysis[2], we have detected γ-Fe_2O_3 (maghemite) *via* Raman as well. Along with maghemite, Raman shifts related to α-Fe_2O_3 (hematite) were also identified. However, hematite could not be detected by air-protected capillary single crystal X-ray diffraction (Supplementary Fig. 18b), which revealed the existence of both γ-Fe_2O_3 and K_2CO_3 on the fresh catalyst as well as a complex mixture of χ-Fe_5C_2, γ-Fe_2O_3 and K_2CO_3 on the spent catalyst. Although we used immersion oil and low laser power during the Raman measurement to prevent the formation of hot spots, the existence of hematite can be explained with the conversion of maghemite-to-hematite *via* laser-induced heating[28–30]. When we analyzed the spent Fe_2O_3@K_2CO_3 *via* Raman spectroscopy, the heterogeneous character of the catalyst was more predominant (Supplementary Fig. 19 and 20). The appearance of carbon D, G, 2D, and D' bands (ca. 1340, 1600, 2650, and 2930 cm^{-1}, respectively)[31] can be considered as the fingerprint of coke formation during the reaction (Supplementary Fig. 19), which has earlier been identified in numerous Fischer-Tropsch Synthesis studies already[32–37]. Herein, χ-Fe_5C_2 is considered the active phase for FTS[2] (also see Supplementary Fig. 18b), which has been covered with amorphous/graphitic hydrocarbon layer during the reaction. Other than the carbon-related Raman vibrations, the existence of Fe_3O_4 on the spent sample was confirmed as well (Supplementary Fig. 19 and 20). Considering the reduction mechanisms reported for Fe_2O_3 to Fe_5C_2 ($Fe_2O_3 \rightarrow Fe_3O_4 \rightarrow Fe_5C_2$), the detection of Fe_3O_4 on the spent sample can be anticipated[38–40]. On the other side, we observed some Raman shifts (ca. 1003 cm^{-1} in Supplementary Fig. 19 (blue cross) – 1004, 1111, 1296, 1431 cm^{-1} in Supplementary Fig. 20) on the spent catalyst which may indicate the presence of CO-containing K structures (i.e., K_2CO_3, $KHCO_3$, KOOCH)[41–43]. It is worth mentioning that all these inorganic K-based carbonylated salts were identified before using by high-field ^{39}K solid-state nuclear magnetic resonance (ssNMR) spectroscopy by us[2].

Herein, we would like to emphasize that no organic carbonylated species have been identified on the metallic Fe-K phase by any means of spectroscopic/characterization tools. Such organic carbonylated species, as illustrated in Fig. 4 in the main text, were detected solely on the zeolite phase.

Zeolite characterization. To illuminate the effect of zeolite only, it is mandatory to alter the zeolite-phase from the Fe_2O_3@KO_2/zeolite-based material, without any alteration at the standalone metal catalyst. An in-depth characterization of standalone Fe_2O_3@KO_2 catalyst has been previously reported by us.[2–5] Herein, eight different zeolites have carefully been selected, considering their versatile properties to build an appropriate database for this comprehensive study (see Supplementary Tables 1, 2). In this database, four 3D Zeolites (ZSM-5, SAPO-34, BETA, and Y), three 2D zeolites (MOR, FER and ZSM-58), and one 1D Zeolite (ZSM-22) were included. Zeolites MOR, BETA and Y have the largest rings with 12 members, while zeolites SAPO-34 and ZSM-58 have only 8 members in the largest ring. Regarding the acidity (silica/alumina) of zeolite, zeolite SAPO-34 has the lowest values (0.5), whereas ZSM-5 and ZSM-22 have the highest values (52 and 65, respectively). The acidity and textural properties of these zeolites were investigated by NH_3-TPD (Temperature Programmed Desorption) and N_2 Physisorption, respectively (see Supplementary Tables 1, 2, and Supplementary Fig. 1). During physisorption analysis, we observed microporosity for all commercial zeolites, except ZSM-22, and surface areas estimated after physisorption are very close to values reported by manufacturers (Supplementary Table 2). Besides these, hysteresis loops observed in some samples (e.g., Y) can indicate the agglomeration of zeolite particles and/or mesoporous structures to some extent (Supplementary Fig. 1a). Since all these zeolites with different topologies and Si/Al ratio were provided by different manufacturers, the amount, distribution, strength, and the proximity of acid sites are expected to

be different in every case and hence, we limited our acidity analysis to NH_3-TPD only in this work (Supplementary Fig. 1b). Ammonia has a relatively small kinetic diameter (ca. 2.6 Å) with strong base properties, which provides high accessibility and strong interactions with acid sites. However, it should be remembered that NH_3-TPD results cannot be directly linked to the intrinsic acidic properties of zeolites.[44] Furthermore, adducts formed between different probes (i.e., strong base NH_3 vs. weak base CO) and different Brønsted acid sites (BAS) can result in distinct destabilizing constraints and dispersion forces.[20] Therefore, each hydrocarbon molecules' interaction with each acid sites in various zeolite topologies can be very discrete under the applied reaction conditions. The amounts of chemisorbed ammonia calculated for commercial zeolites are presented in Supplementary Table 2. The ammonia desorption curves of the zeolites are the interesting facet of these analyses. Except for ZSM-5 and FER, which have two maxima points, for all other zeolites, one main broad desorption peak was observed during the TPD experiments, mostly being lied between 300 °C and 500 °C (Supplementary Fig. 1b), indicative of typical strong BAS. If we only consider the peaks above 300 °C, BETA and Zeolite-Y have the weakest ammonia adsorption strengths (ca. 349 and 337 °C, respectively). On the other hand, acid sites on MOR revealed the strongest interaction with ammonia molecules (maxima ca. 505 °C). The trend we obtained from maxima points (MOR>>FER>ZSM-22≥ZSM-58≥ZSM-5≥SAPO-34>>BETA>Zeolite-Y) are pretty matching with the NH_3 desorption enthalpies reported in the literature.[45] Also, the strong interactions of MOR with ammonia molecules might be related to the acid sites located in the 8 MR.[45] In general, when the amount of chemisorbed ammonia is calculated for commercial zeolites, it is clear that amounts are correlated with SiO_2/Al_2O_3 for all samples, except ZSM-5 and ZSM-58. Moreover, both ZSM-5 and FER showed an additional peak maximum at <300 °C, which symbolizes weak acid sites (Supplementary Fig. 1b). As it is known that some reactions demand

low acidic strength;[46–48] it can be speculated that in this system, acid sites forming weak adducts on ZSM-5 and Ferrierite might be the reason for the significant change in hydrocarbon distributions. Since BAS originate from the bridging hydroxyl groups between Al and Si atoms, ^{27}Al MAS ssNMR spectroscopy has also been performed on selected zeolites (ZSM-5, FER, MOR, ZSM-22) to probe the local-Al environment, which could be correlated to the acidic properties (Supplementary Fig. 2). All samples demonstrated a similar spectral pattern, where a peak around ~52 ppm indicates tetrahedral framework-Al. Both penta- (~27 ppm) and octahedral (~-3 ppm) could only be observed for FER and MOR, suggests the presence of Lewis acidic sites (LAS).[3,49] In the post-reacted zeolite materials, octahedral LAS were present on all samples, except ZSM-22. However, in MOR, the extent of LAS is appeared to be relatively decreased after the reaction. Such an 'alumination-dealumination' feature could be assisted by Reverse Water-Gas Shift Reaction (RWGS)-derived water during the actual reaction condition ($CO_2 + H_2 \rightleftharpoons CO + H_2O$, see Fig. 7 in the manuscript).

Relevance to catalysis. Although SAPO-34 and ZSM-58 have strong interactions with NH_3 molecules, those corresponding acid sites may not be accessible for the relatively bigger molecules (i.e., C_{2+}). On the contrary, the weak interactions of zeolite-Y (FAU) with ammonia means that the bigger void structures are not able to fulfill the necessary requirement (confinement, dispersive forces etc.). In addition to the desorption peaks in the range mentioned above, ZSM-5 and FER showed additional desorption peaks having maxima smaller than 300 °C (ca. 241 and 256 °C, respectively). It is a known fact that some reactions may demand weaker interactions with acid sites and their located void. So, these sites in ZSM-5 and FER having weaker NH_3 adsorption strength might be one of the key contributors to the significant change in hydrocarbon distributions and specifically promoting hydrogen transfer activity (Fig. 2b). As it is clearly seen from

Supplementary Table 2; while nitrogen (or ammonia) molecule can diffuse inside the pores of some zeolites (e.g., Y) and be adsorbed on the surface easily, for some zeolites (e.g., ZSM-22), it cannot access all available pores and cages, most likely due to defects in the sample (i.e., kinetic diameters of N_2 and NH_3: 3.72 Å and 2.6 Å, respectively). This also means that in a dual-bed system, some components formed on Fe_2O_3@KO_2 cannot diffuse directly inside the pores-cages of some zeolites and interact with catalytic sites inside (i.e., kinetic diameters of C_2H_4 and CO: 3.90 Å and 3.76 Å, respectively). Furthermore, it is always important to consider the heterogeneity of acidities even within the same zeolite structure (i.e., the location of acid sites in pores-cages having different dimensions, pointing directions of protons, and probe molecules' intrinsic features). Therefore, adducts formed between different probes (i.e., strong base NH_3 vs. weak base CO) and different BAS can result in dissimilar destabilizing constraints and dispersion forces.[20] This heterogeneity feature for all zeolites has clearly been illuminated in our ssNMR spectroscopic-based mechanistic study.

Extended mechanistic discussion and solid-state NMR analysis. To verify the assignments of the oxygenated species in this work (i.e., both carbonyl and methoxy region), several control 1D ^{13}C-based ssNMR experiments were performed (Supplementary Fig. 12-13). Initially, ^{13}C DE ssNMR spectra were performed on several organic compounds chemisorbed on zeolites. In a typical experiment, NMR rotors were packed with the zeolite and followed by the addition of the required amount of organic compounds. NMR rotor caps were kept open to evaporate the excess liquid, if any. After that, the spectra have been taken using a long recycle delay of 10 s, 16 ms acquisition time, and accumulation of 4k scans. The following chemical shifts were observed in respective ^{13}C DE spectra: (i) acetone on zeolite FER: 207.5 ppm ($CH_3\underline{C}OCH_3$) and 30.7 ppm ($\underline{C}H_3CO\underline{C}H_3$), (ii) methyl formate on zeolite MOR: 164.0 ppm ($H\underline{C}OOCH_3$) and

51.4 ($HCOO\underline{C}H_3$), (iii) diacetyl on zeolite ZSM-5: 198.4 ppm ($(CH_3\underline{C}O)_2$) and 23.4 ppm ($(\underline{C}H_3CO)_2$), (iv) methanol on ZSM-5: 50.0 ppm (CH_3OH), and (v) methyl acetate on ZSM-22: 171.6 ppm ($CH_3\underline{C}OOCH_3$), 51.4 ppm ($CH_3COO\underline{C}H_3$), 20.2 ppm ($\underline{C}H_3COOCH_3$). These peak positions clearly confirm our original assignments in Figs. 4e,5f,6d on post-reacted zeolites: (i) acetone on FER: 211.9 ppm ($CH_3\underline{C}OCH_3$) and 29.8 ppm ($\underline{C}H_3CO\underline{C}H_3$), (ii) diacetyl on ZSM-22: 198.9 ppm ($(CH_3\underline{C}O)_2$) and 24.1 ppm ($(\underline{C}H_3CO)_2$), (iii) diacetyl on ZSM-5: 197.5 ppm ($(CH_3\underline{C}O)_2$) and 23.0 ppm ($(\underline{C}H_3CO)_2$), (iv) methanol on ZSM-5: 52.1 ppm (CH_3OH), (v) surface acetate on ZSM-22: 180.2 ppm ($CH_3\underline{C}O$-zeolite), 18.7 ppm ($\underline{C}H_3CO$-zeolite), (vi) surface acetate on FER: 176.6 ppm ($CH_3\underline{C}O$-zeolite), 19.2 ppm ($\underline{C}H_3CO$-zeolite), and (vii) methyl acetate on FER (cf. control experiment): 177.5 ppm ($CH_3\underline{C}OOCH_3$), 50.7 ppm ($CH_3COO\underline{C}H_3$), 18.4 ppm ($\underline{C}H_3COOCH_3$).[6,50–52] It would be worth clarifying that surface acetate species, not methyl acetate, has been assigned on post-reacted FER and ZSM-22 after the reaction, due to the absence of correlation between the carbonyl carbon and a methoxy carbon (Fig. 4e). Contrary, this correlation has clearly been detected on the post-reacted FER after the control experiment (Fig. 6d) and hence, this assignment has been attributed to the methyl acetate. Next, ^{13}C DE ssNMR of several potassium-based salts (K_2CO_3, $KHCO_3$, HCO_2K, and KO_2+CO_2) have also been recorded (Supplementary Fig. 13). Although these potassium-salts were involved in the potassium-phase of our metallic catalyst (Fe_2O_3@KO_2) (see Fig. 7),[2] none of these peak positions matched with any carbonylated species on all four post-reacted zeolites. Herein, no potassium-based salts were transferred from the metallic phase to the zeolite-phase and thus, all carbonyl-based peaks on our post-reacted zeolites were purely organic in nature. All these carbonylated species are interconvertible among themselves and, interestingly, have a very high mechanistic resemblance with the established combustion chemistry of ketene from literature (see Supplementary Fig.

24).[53–55] Herein, ketene is the only *missing link* among other intermediates: acetate is a physisorbed ketene with an energy gain of >50 kJ/mol and a minimal energy barrier of ≤17 kJ/mol[56,57], as well as ketene is reversibly accessible from both acetone[58] (via Schmidlin synthesis) and diacetyl[53–55] at high-temperature (see Supplementary Fig. 24). It should be worth mentioning that CO of ketene is not consumed in the reaction product, neither incorporated itself in the HCP species[7,57]. It should be worth mentioning that none of these organic carbonyls, *to the best of our knowledge*, neither spectroscopically verified nor proposed in this chemistry before. Moreover, we should also acknowledge that some of these intermediates were already reported during the electrochemical reduction of CO_2.[59–65] Herein, particularly the proposed "CO-dimerization pathway" (*i.e.*, a way to produce C_{2+} molecule electrochemically from CO_2[59–61]) during electrochemical CO_2 recycling indeed have mechanistic resemblance with our current thermocatalytic CO_2 hydrogenation system. It should also be emphasized that such dimerization of CO could indeed proceed through a non-electrochemical pathway as well,[63–65] and hence, bears mechanistic similarity with the current work. In essence, considering all above arguments, we must acknowledge the co-catalytic role of CO in this zeolite chemistry (Figure 6 & Supplementary Fig. 14).[52,66]

The effect of mobility has scarcely been highlighted while describing zeolite catalysis.[6,67–70] The mechanistic study presented in this work clearly revealed that the mobility features, or the host-guest interaction in general, governing the formation of reaction intermediates and products during the catalysis. Strategically, two following approaches have been applied to investigate the mobility of zeolite-trapped species: to probe mobile or rigid ^{13}C-^{1}H correlations, 'through-bond' scalar or 'through-space' dipolar magnetization transfer techniques, respectively, was used to polarize the carbons, whereas in the ^{13}C-^{13}C correlation spectra, the ^{13}C-carbons were polarized

through DE (for all species) or CP (for rigid species) and ^{13}C-^{13}C mixing was achieved through proton-driven spin-diffusion (PDSD) using phase-alternated recoupling irradiation schemes (PARIS). For example, in this work, both MOR and ZSM-5-trapped species typically demonstrated comparatively rigid behavior, while FER pose the highest volume of sp^3-species; thus, predominantly mobile in nature. Contrary, ZSM-22 appeared to have a balanced mobility feature, where C_5-C_9 olefins were preferred. In general, the broad nature of CP-based measurements is quite common for all four zeolites (Supplementary Figs.4,5), particularly in the C_{sp2}-regions, that could be attributed to their rigidity/restricted mobility nature. Sometimes, it also affected the respective cross-peak between 'rigid' aromatics/olefins and relatively more 'mobile' aliphatic methyl groups. For instance, FER primarily consisted of a high volume of mobile C_{sp3}-aliphatic species along with minor rigid C_{sp2}-species, which has led to a lack of cross-peaks between them (Supplementary Figs. 6,7,10). We anticipate that such behavior from the zeolite-trapped rigid species could be correlated to their quick decay of CP-based signals. This phenomenon could further be connected to the transverse (or spin-spin) relaxation time (T_2), which is one of the processes for the deterioration of the NMR signal, and directly is responsible for the broadening of the signal. Since the rigid species, by default, have very short T_2, which directly corroborated to the broad linewidth (linewidth $\propto 1/T_2$), as also observed in the present study. Therefore, the host-guest chemistry indeed crucial within the zeolite-pores. Moreover, this is another important factor to consider. It should be noted that the overall correlation pattern in CP-based ^{13}C-^{13}C experiments exhibits asymmetry relative to the main diagonal (Supplementary Fig. 5). This feature (not visible in the DE-based 2D ^{13}C-^{13}C experiments, see Supplementary Figs. 8-11) echoes the fact that cross peak intensities reflecting transfer from (more)-protonated to less/non-protonated spin species are much stronger than the reverse path. However, the latter

correlations are readily observable in DE-based correlation experiments.[52] Therefore, from 2D ^{13}C-^{13}C experiments, the information related to the mobile species (including intermediate dynamics) were extracted from the combination between DE-based 2D ^{13}C-^{13}C experiments and INEPT HETCOR/HSQC based ^{13}C-^{1}H correlation experiments (Supplementary Figs. 6-11), while all CP-based measurements were entirely dedicated to the rigid species only (Supplementary Figs. 4,5).

The above-mentioned mobility features and host-guest chemistry between inorganic zeolite and organic species have been extended to the catalysis (or *vice-versa*). Fig. 2 and Fig 6a-b in the manuscript clearly reveal that the formation of light olefins was indeed increased at the expense of consumption of CO for most zeolites, specifically in MOR, yet, no other carbonylated intermediates were detected by ssNMR spectroscopy. Also, MOR-based responses in ssNMR spectroscopy were exceptionally broad, particularly in the C_{sp2}-hydrocarbon regions (Supplementary Figs. 4,5b), which indicates that MOR-trapped species were rigid in nature, presumably these were in the form of π-complexes on BAS of zeolite and hence, restricting mobility.[68] Therefore, it is safe to assume that CO is directly responsible for the formation of short olefins (Fig 6a-b). After MOR, high CO consumption has been observed for FER, where paraffins is the primary product (Fig. 2 in the manuscript). But contrary to MOR, multiple other carbonylated species (surface acetate and acetone) were detected in FER (Supplementary Fig. 10), which might be connected to their mobility and topological characteristics. Next, both ZSM-5 and ZSM-22 demonstrated almost similar CO consumption, which is lesser than MOR or FER (Fig. 2 in the manuscript). Among all carbonylated species, diacetyl has been detected on both ZSM-5 and ZSM-22, while ZSM-22 shows an additional signal for surface-acetate (Fig. 4e). In ZSM-5, aromatics were the primary product and (like MOR) contains more rigid species (Supplementary Figs. 4,5a). Therefore, it is worth emphasizing that rigidity characteristics were more predominant in the

presence of aromatics or olefinic products, presumably due to their existence in the form of π-complexes on BAS of zeolites.[68,70] Next, in ZSM-22, when the product is long-chain hydrocarbons/olefins, it shows more intermediate dynamics, due to the presence of long chain aliphatic moieties (hence, relatively more mobile, like FER) and olefin groups (hence, more rigid, like MOR) in a single-molecule. An almost similar trend of CO-consumption has also been noted in the control experiments (MOR>FER>ZSM-5~ZSM-22, see Fig. 6b in the manuscript). However, due to the selective isotope enrichment on ^{13}CO only (cf. along with the natural-abundance ethylene), only CP-based HETCOR experiments were feasible in all cases (Supplementary Figs. 16). However, surprisingly, despite having lower CO consumption than MOR, excellent DE-based responses were detected from the zeolite FER only (Supplementary Fig. 17). Consistent with our previous observation after the usual reaction, zeolite FER contains mobile species mostly after the control experiment too. As highlighted earlier, the transverse (or spin-spin) relaxation time (T_2) is also an important parameter here. Therefore, it is safe to correlate higher mobility to better signal-to-noise ratio, which is mainly predominated by the paraffinic products.

Extended computational analysis and discussion. Adsorption or bond energies of various species adsorbed on different zeolite species are displayed in Table 1 and additional statistical analysis are included in Supplementary Table 3. Further evidence to support 2D NMR observations is that H-ZSM-22 and H-FER frameworks have a higher surface-acetate bonding energy, with ~30 kJ/mol difference from the zeolites in which surface-acetate was not detected. Since H-ZSM-22 and H-ZSM-5 have the same deprotonation energies,[14] charge transfer effects do not fully explain framework surface-acetate stabilization.

H-MOR [T1] has a similar CO adsorption strength to the other analyzed active sites; the significant difference in bonding capability is evidenced when comparing the values of the other organic carbonylates. The higher instability of carbonylates in H-MOR [T1], with H-MOR [T1] having a 5 to 20 kJ/mol weaker adsorption strength compared to the rest of the zeolites, would make the reaction equilibrium lean towards CO to a higher degree than the other active sites.

Ketene has a small difference (-3 to 12 kJ/mol) in adsorption strength, depending on the orientation to the zeolite acid site (as illustrated in Supplementary Fig. 23), thus ensuring a wider scope of reaction routes. This outcome could be due to the symmetric structure of ketene, which makes the electronic density to be evenly distributed. Since ketene bonding is slightly more exothermic via the carbon center adsorption, there is a favorable steric configuration to surface-acetate formation by ketene reaction with the Brønsted proton.[52,57,66,71] However, CO insertion is the primary reaction route to surface-acetate, based on the energetics involved. Specifically, CO insertion to methoxy (93 kJ/mol) is approximately twice more exothermic than ketene conversion (~40 kJ/mol). The main difference in the surface-acetate formation energies, when comparing the two conversion routes, may be determined by the fact that ketene undergoes a significant skeletal rearrangement, than methyl or CO in the case of CO insertion, in addition to the types of bonds being broken in the initial stage of the reaction. The most exothermic reaction energies to form surface-acetate from ketene are encountered in H-ZSM-5 [T12], [T6] and H-FER [T1], which would lead to surface-acetate being detected in these two zeolites.

Theoretical investigations indicated the possibility of a competitive adsorption equilibrium between acetone, methyl-acetate, and diacetyl, with a proposed outlook detailed in Supplementary Fig. 25. Since there is similar stability of the aforementioned intermediates, the selective experimental observation may indicate that diffusion or confinement effects could influence the

accumulation of certain organic carbonylates more than others. The additional geometric analysis presented in Supplementary Fig. 21-23, could not uncover any structure adsorption relations, as previously reported.[20,72]

2D NMR detected acetone only in H-FER. This suggests high reactivity of acetone inside the zeolite pores. Contrasting observations can be made in the case of diacetyl and methyl-acetate. Based on the adsorption energies represented in Table 1, the stability of diacetyl and methyl-acetate is comparable in all zeolites, while experiments found methyl acetate only in H-ZSM-22 and H-FER, suggesting higher reactivity for methyl acetate in H-ZSM-5 and H-MOR. The 2D NMR carbonylated species detected in the 2D pore zeolites found that CO (in H-MOR [T1]) and acetone (in H-FER [T1]) are present inside the channels, with intermediates such as diacetyl or methyl-acetate being absent. Although H-FER has a slightly smaller pore size than H-ZSM-22, the 2D pore network may allow for a more even distribution of reactants and diffusion of acetone, limiting the formation of diacetyl. Ketene has an adsorption energy ~30 kJ/mol stronger than the rest of the analyzed carbonylates, thus being able to substitute each carbonylate on the adsorption site. Since there is no preferred adsorption orientation, if the acid sites are close to each other, one ketene molecule could bond and bridge both sites, thus potentially blocking the zeolite pores and preventing other intermediates to access the active sites.

To summarize, computational analysis validated 2D NMR observations by showing that surface-acetate has higher stability on H-ZSM-22 and H-FER. H-MOR is more likely to have CO adsorbed because the additional carbonylates are more unstable on H-MOR acid sites than other zeolites investigated. Ketene can be involved in surface-acetate formation. However, thermodynamically, the dominant reaction path is via CO insertion to methyl. Further analysis focused on diffusion effects may be necessary to understand the selective observation of different

organic carbonylates in each zeolite. Apart from shape selectivity being a key factor in the production of hydrocarbons, the pore's structure may also influence the initial stages of the reaction process.

Supplementary Figures

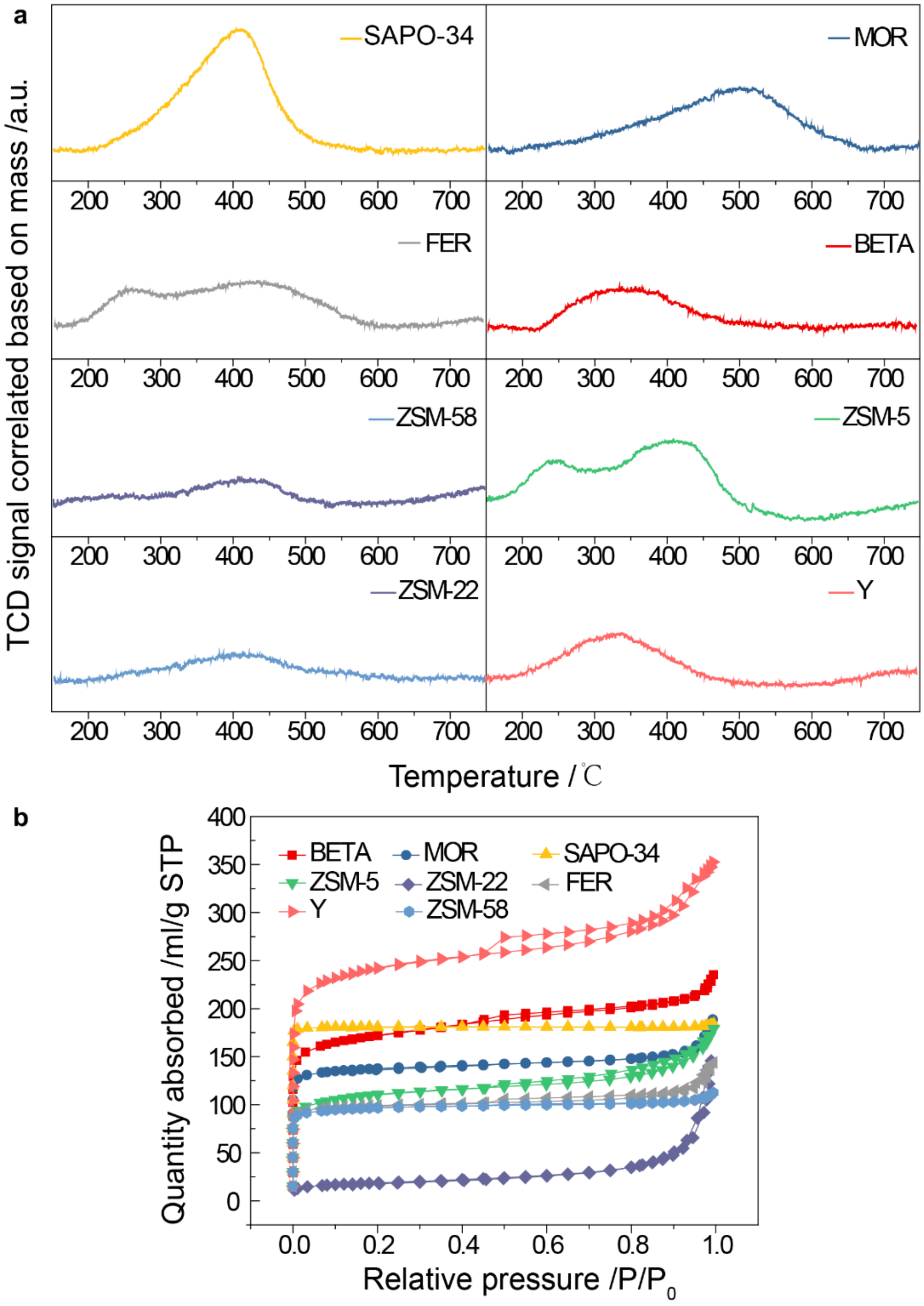

Supplementary Fig. 1 Characterization of zeolites. (a) NH_3-TPD profiles and (b) N_2 adsorption isotherms of all zeolites.

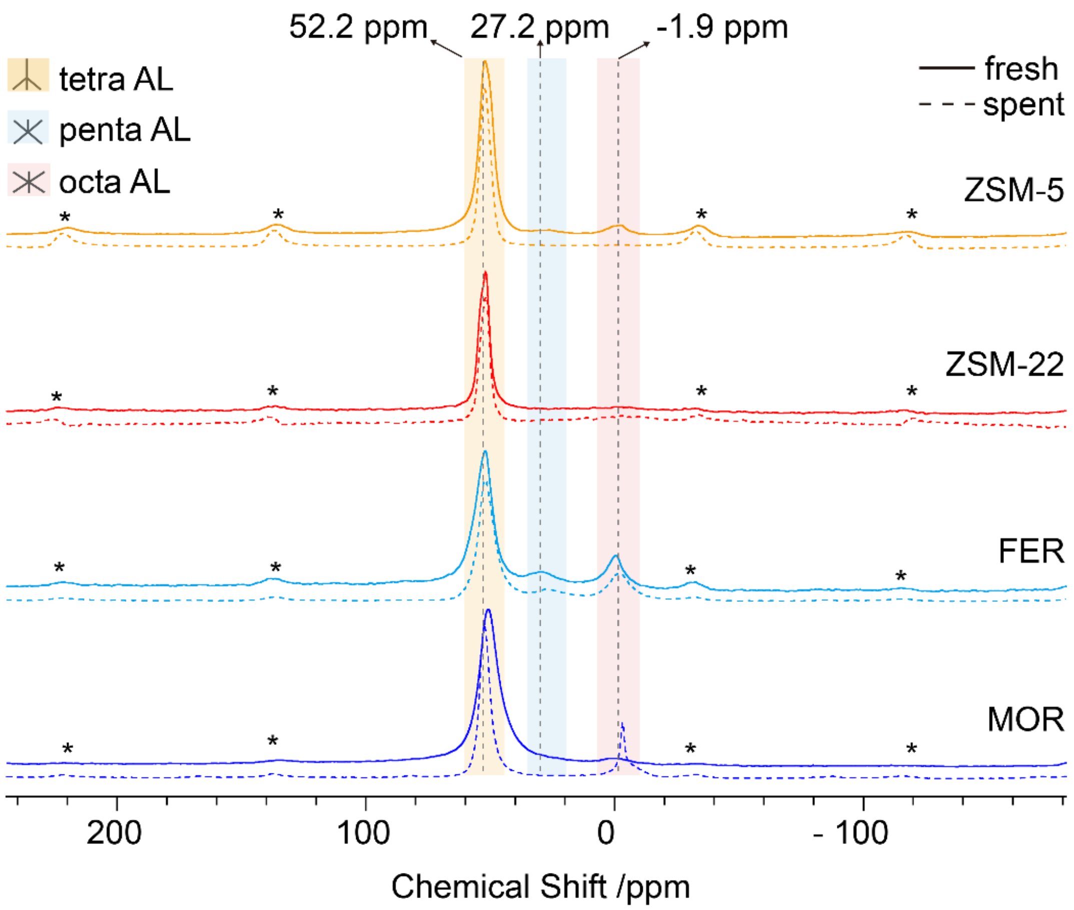

Supplementary Fig. 2 ^{27}Al ssNMR spectra of fresh (—) and spent (---) zeolites ZSM-5, ZSM-22, FER, and MOR (20 KHz magic angle spinning, *=spinning sideband). Spectra of post-reacted zeolites were obtained after the hydrogenation of fully isotope-enriched $^{13}CO_2$ in the reactant feed ($^{13}CO_2$ at 30 bar, 375ºC, $H_2/^{13}CO_2$=3, and 10000 mL·g^{-1}·h^{-1} at a time-on-stream of 48 hours).

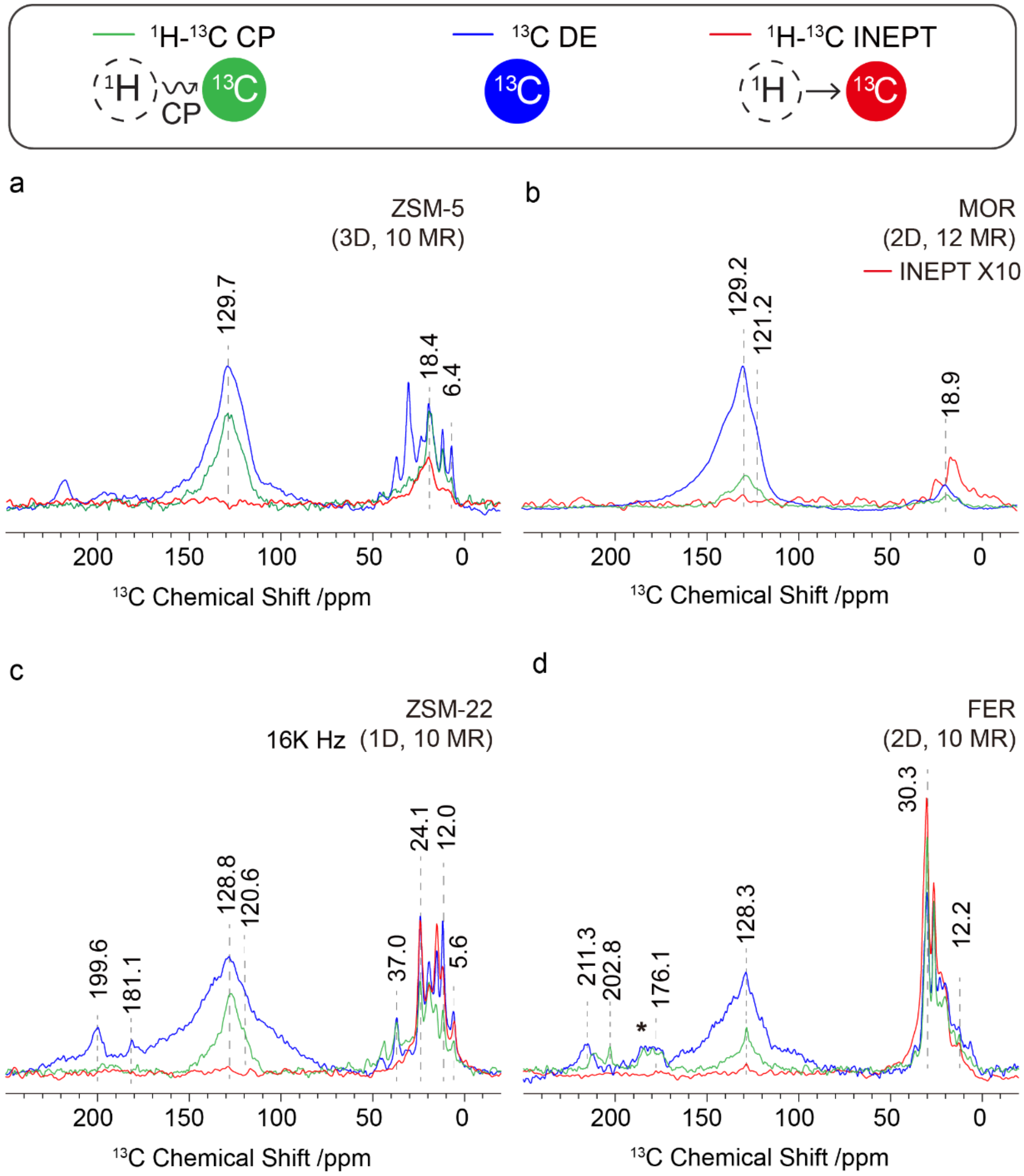

Supplementary Fig. 3 1D ^{1}H-^{13}C CP (green), ^{13}C DE (blue) and ^{1}H-^{13}C INEPT (red) MAS ssNMR spectra of post-reacted zeolites: (a) ZSM-5, (b) MOR, (c) ZSM-22, and (d) FER (MAS: magic angle spinning (20KHz for ZSM-22 c and 16KHz for others), *=spinning sideband). Spectra of trapped products obtained on respective post-reacted zeolites after the hydrogenation of fully isotope-enriched $^{13}CO_2$ in the reactant feed ($^{13}CO_2$ at 30 bar, 375ºC, $H_2/^{13}CO_2$=3, and 10000 $mL\cdot g^{-1}\cdot h^{-1}$ at a time-on-stream of 48 hours).

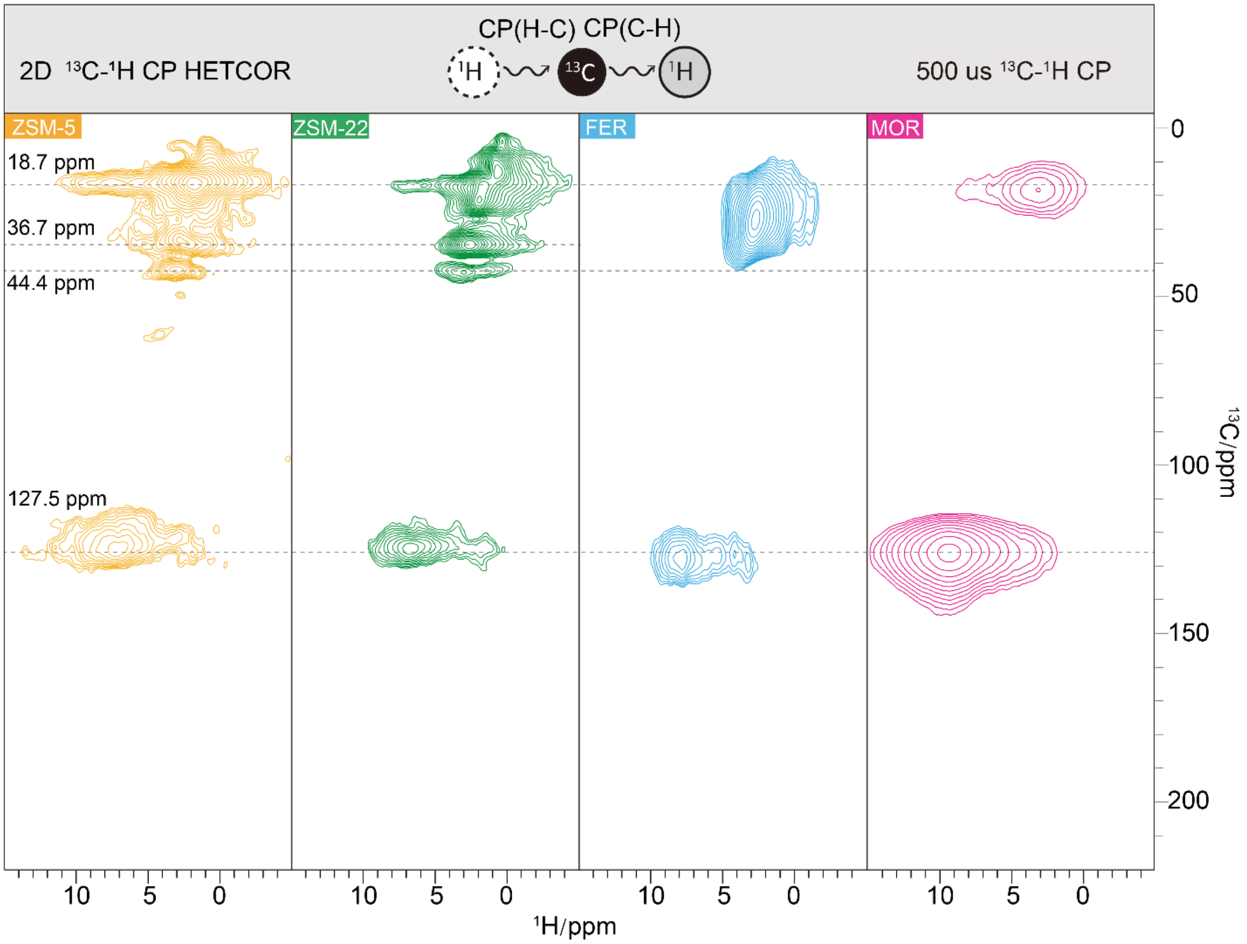

Supplementary Fig. 4 2D ^{13}C-^{1}H CP-based MAS ssNMR correlation spectra of post-reacted four zeolites trapped species to probe rigid molecules (MAS: magic angle spinning (20KHz), CP: cross-polarization, ^{1}H-^{3}C CP contact time: 500μs, p16: ^{13}C-^{1}H CP contact time: 500μs). Spectra of trapped products obtained on respective post-reacted zeolites after the hydrogenation of fully isotope-enriched $^{13}CO_2$ in the reactant feed ($^{13}CO_2$ at 30 bar, 375ºC, $H_2/^{13}CO_2=3$, and 10000 $mL\cdot g^{-1}\cdot h^{-1}$ at a time-on-stream of 48 hours).

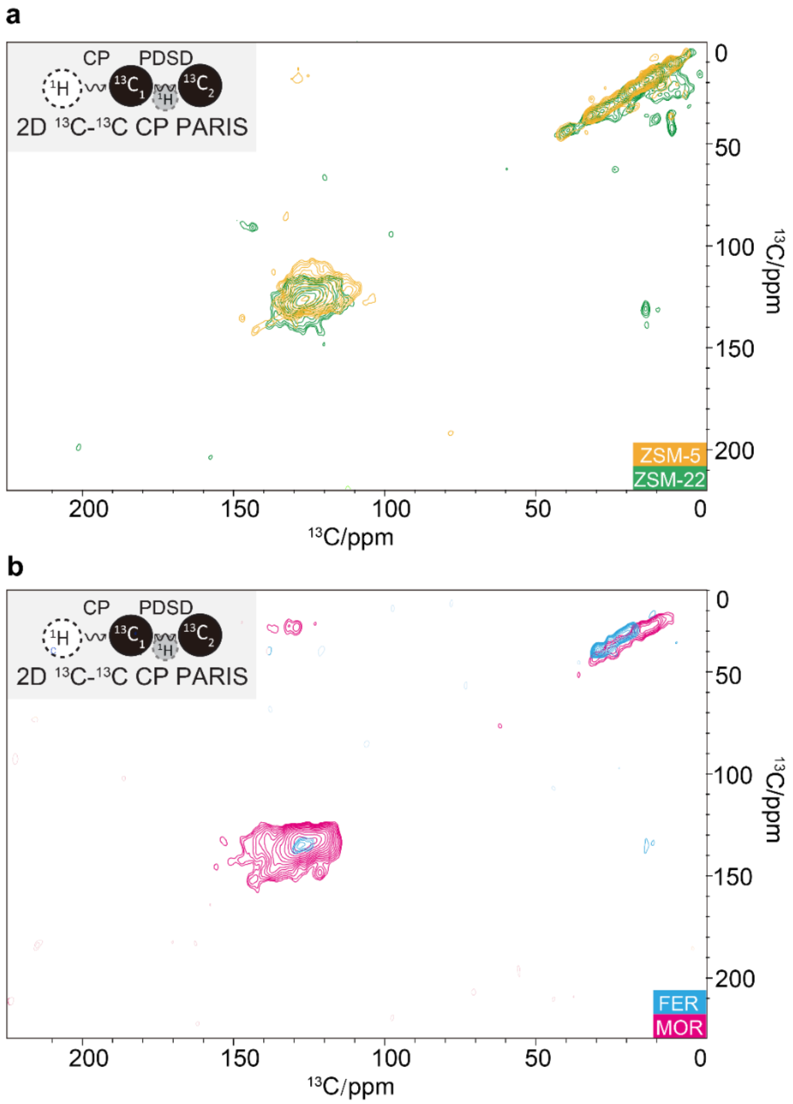

Supplementary Fig. 5 2D ^{13}C-^{13}C CP-based proton-driven spin-diffusion (PDSD), using phase-alternated recoupling irradiation schemes (PARIS), MAS ssNMR correlation spectra of zeolite-trapped species to probe rigid molecules: (a) ZSM-5 and ZSM-22 as well as (b) FER and MOR. Herein, ^{13}C atoms were polarized by ^{1}H-^{13}C CP and a 120ms PARIS mixing period was used (MAS: magic angle spinning (20KHz), CP: cross-polarization). Spectra of trapped products obtained on respective post-reacted zeolites after the hydrogenation of fully isotope-enriched $^{13}CO_2$ in the reactant feed ($^{13}CO_2$ at 30 bar, 375°C, H_2/$^{13}CO_2$=3, and 10000 mL·g^{-1}·h^{-1} at a time-on-stream of 48 hours).

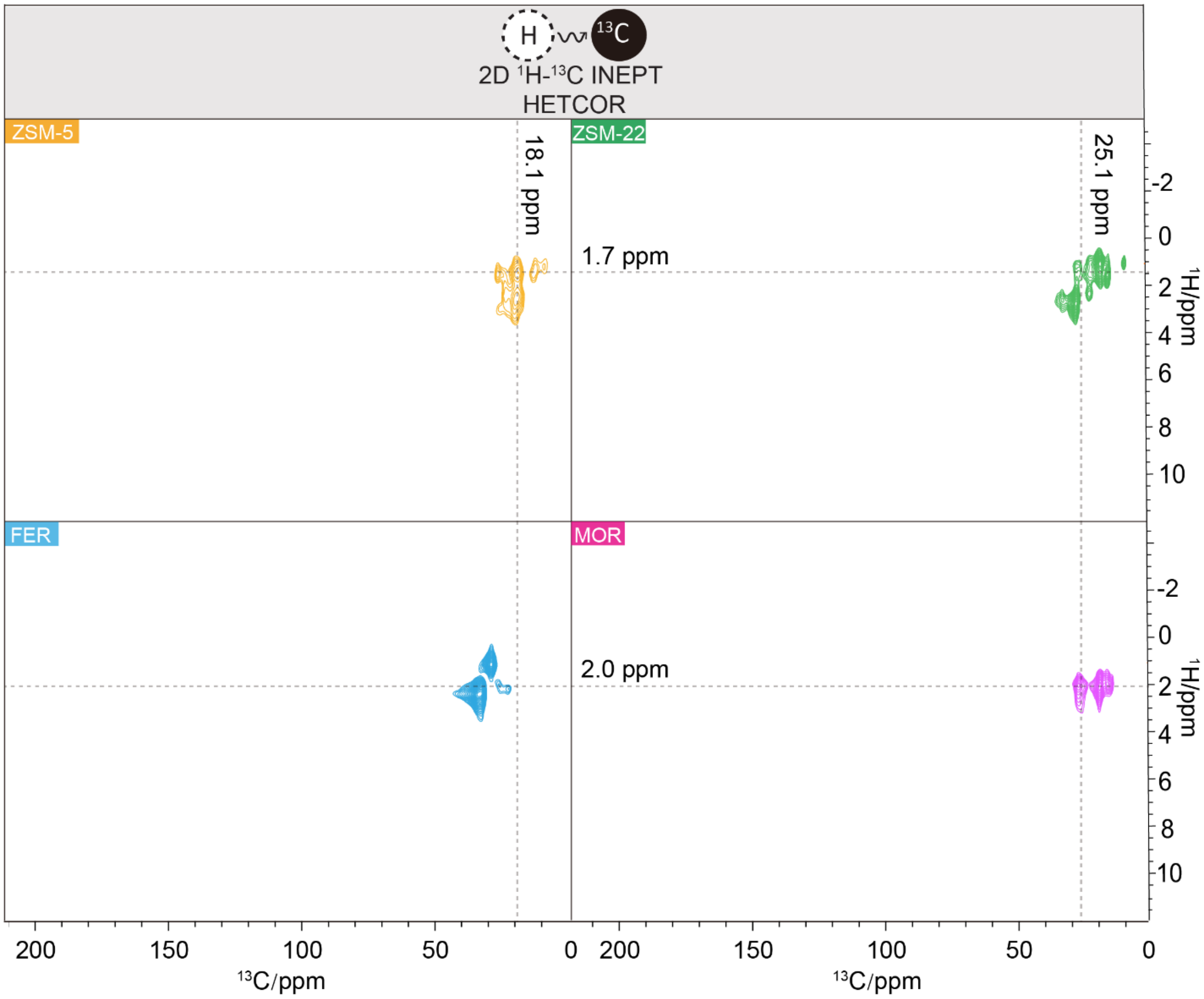

Supplementary Fig. 6 2D ^{1}H-^{13}C INEPT-based HETCOR MAS ssNMR correlation spectra of post-reacted four zeolites trapped species to probe mobile molecules (MAS: magic angle spinning (20KHz), INEPT: Insensitive Nuclei Enhancement by Polarization Transfer, HETCOR: HETeronuclear CORrelation Spectroscopy). Spectra of trapped products obtained on respective post-reacted zeolites after the hydrogenation of fully isotope-enriched $^{13}CO_2$ in the reactant feed ($^{13}CO_2$ at 30 bar, 375°C, $H_2/^{13}CO_2$=3, and 10000 mL·g^{-1}·h^{-1} at a time-on-stream of 48 hours).

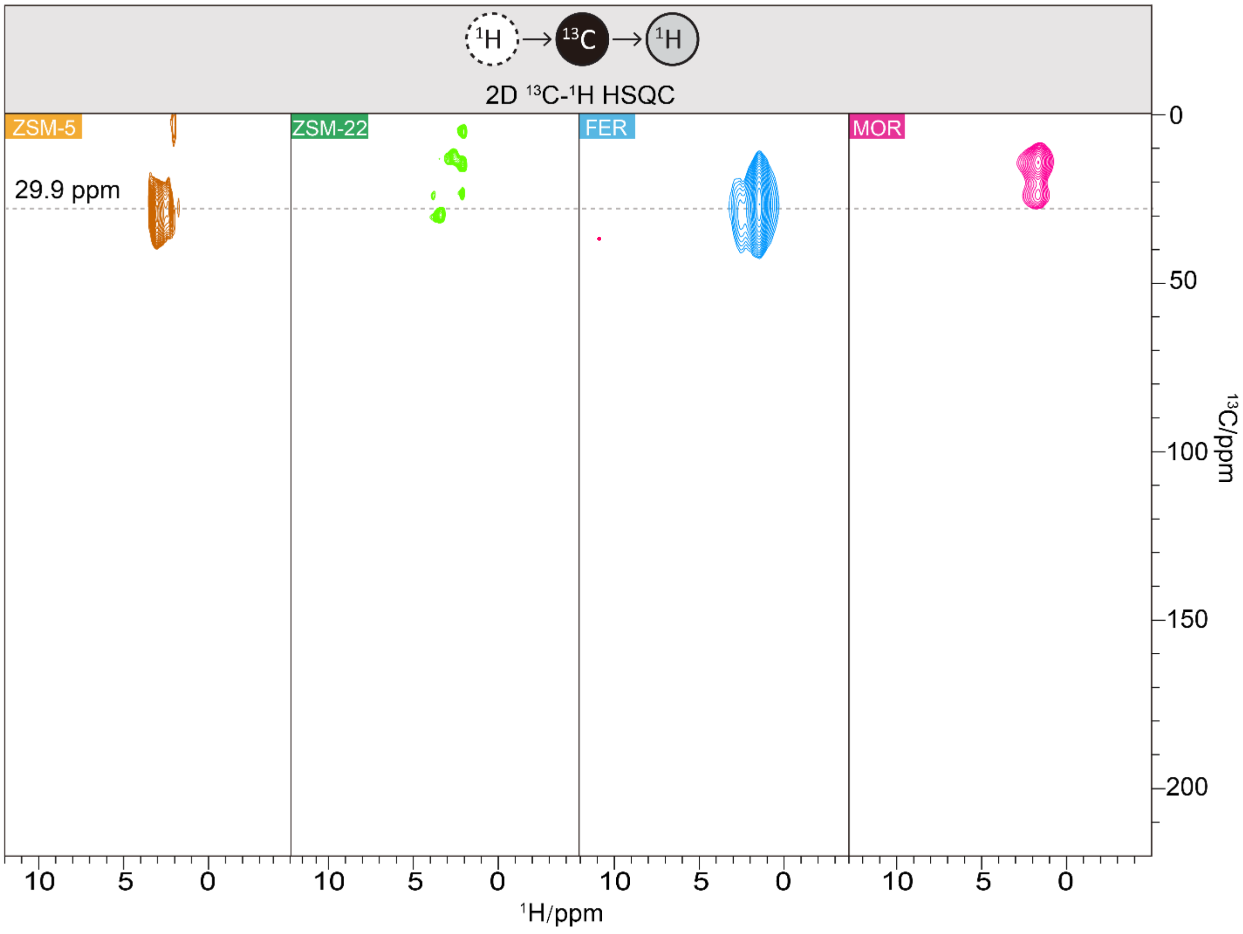

Supplementary Fig. 7 2D ^{13}C-^{1}H HSQC ssNMR correlation spectra of post-reacted four zeolites trapped species to probe mobile molecules (MAS: magic angle spinning (16KHz), HSQC: Heteronuclear single quantum coherence spectroscopy). Spectra of trapped products obtained on respective post-reacted zeolites after the hydrogenation of fully isotope-enriched $^{13}CO_2$ in the reactant feed ($^{13}CO_2$ at 30 bar, 375ºC, $H_2/^{13}CO_2$=3, and 10000 mL·g^{-1}·h^{-1} at a time-on-stream of 48 hours).

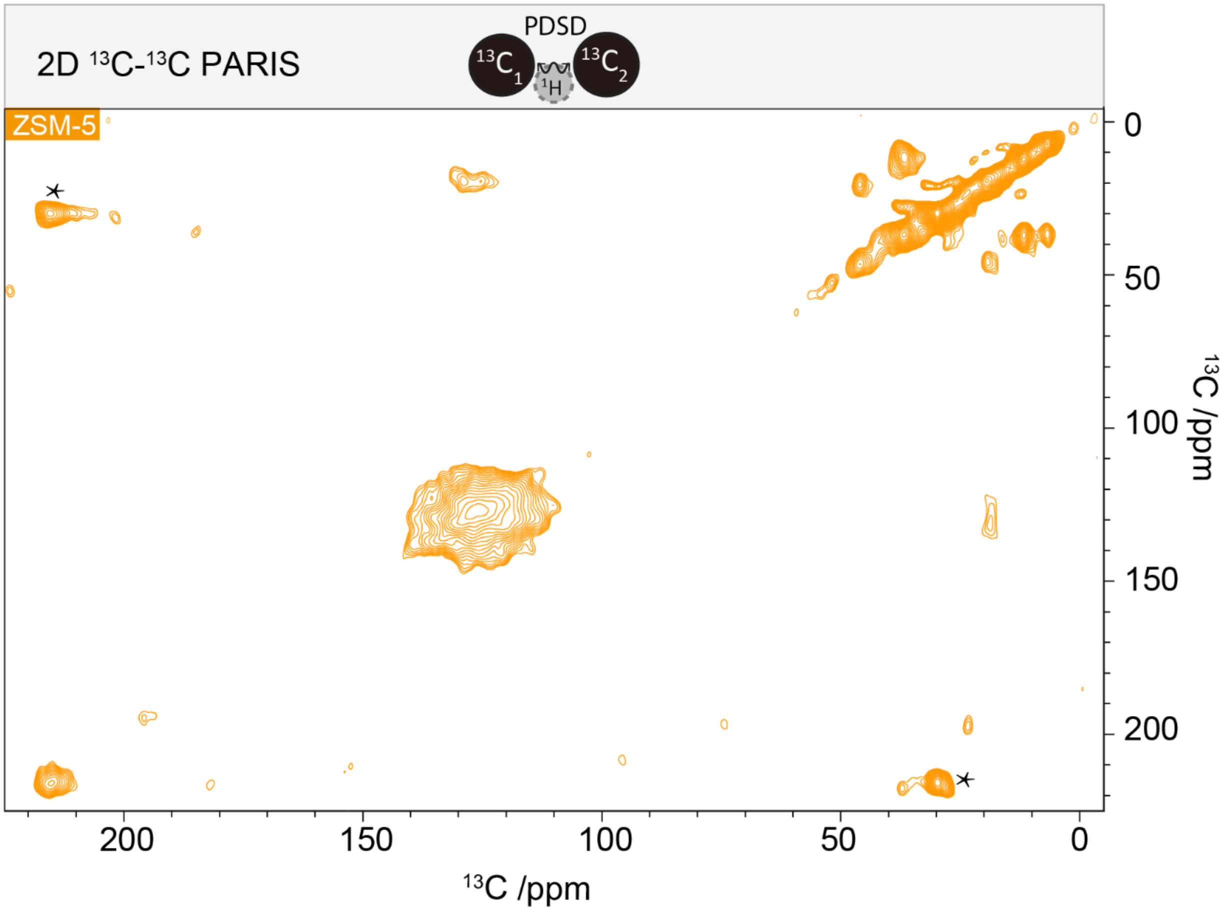

Supplementary Fig. 8 2D ^{13}C-^{13}C DE-based proton-driven spin-diffusion (PDSD), using phase-alternated recoupling irradiation schemes (PARIS), MAS ssNMR correlation spectra of zeolite ZSM-5 trapped species to probe all molecules. Herein, ^{13}C atoms were polarized by DE, and a 200 ms PARIS mixing period was used (MAS: magic angle spinning (16KHz), DE: direct excitation, *: spinning side bands). Spectra of trapped products obtained on respective post-reacted zeolites after the hydrogenation of fully isotope-enriched $^{13}CO_2$ in the reactant feed ($^{13}CO_2$ at 30 bar, 375°C, $H_2/^{13}CO_2$=3, and 10000 mL·g^{-1}·h^{-1} at a time-on-stream of 48 hours).

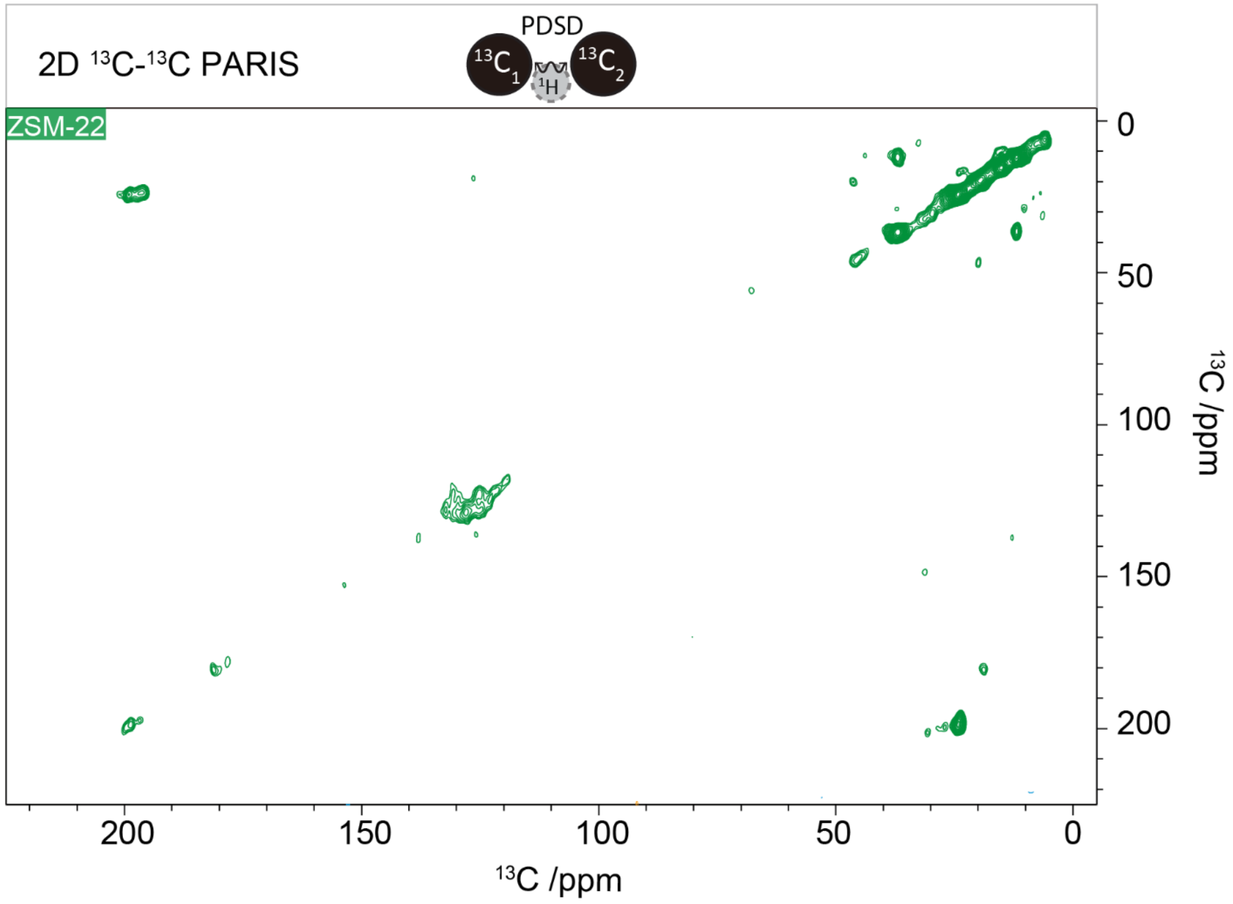

Supplementary Fig. 9 2D ^{13}C-^{13}C DE-based proton-driven spin-diffusion (PDSD), using phase-alternated recoupling irradiation schemes (PARIS), MAS ssNMR correlation spectra of zeolite ZSM-22 trapped species to probe all molecules. Herein, ^{13}C atoms were polarized by DE, and a 200 ms PARIS mixing period was used (MAS: magic angle spinning (20KHz), DE: direct excitation, *=spinning sideband)). Spectra of trapped products obtained on respective post-reacted zeolites after the hydrogenation of fully isotope-enriched $^{13}CO_2$ in the reactant feed ($^{13}CO_2$ at 30 bar, 375ºC, H_2/$^{13}CO_2$=3, and 10000 mL·g^{-1}·h^{-1} at a time-on-stream of 48 hours).

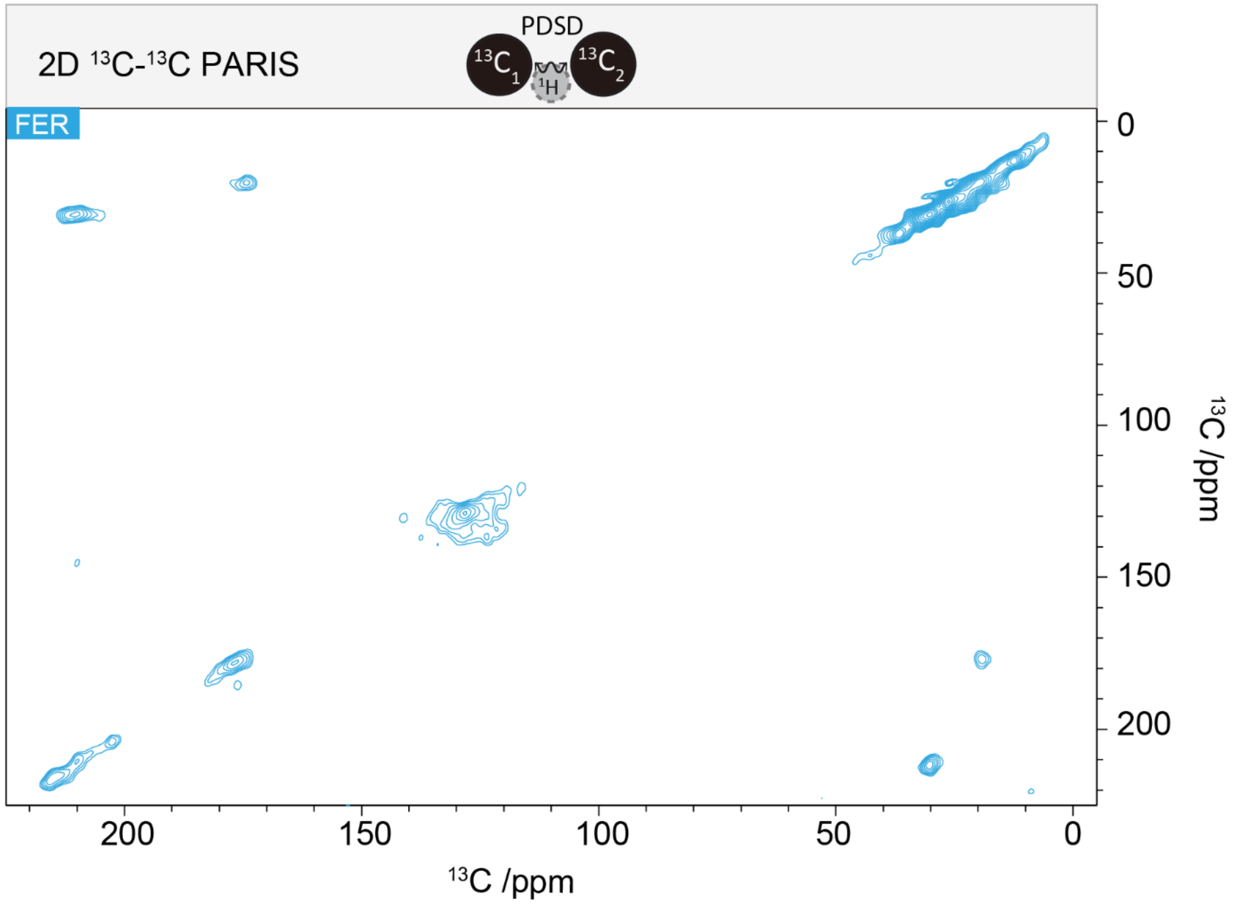

Supplementary Fig. 10 2D ^{13}C-^{13}C DE-based proton-driven spin-diffusion (PDSD), using phase-alternated recoupling irradiation schemes (PARIS), MAS ssNMR correlation spectra of zeolite FER trapped species to probe all molecules. Herein, ^{13}C atoms were polarized by DE, and a 200 ms PARIS mixing period was used (MAS: magic angle spinning (16KHz), DE: direct excitation). Spectra of trapped products obtained on respective post-reacted zeolites after the hydrogenation of fully isotope-enriched $^{13}CO_2$ in the reactant feed ($^{13}CO_2$ at 30 bar, 375ºC, $H_2/^{13}CO_2$=3, and 10000 mL·g^{-1}·h^{-1} at a time-on-stream of 48 hours).

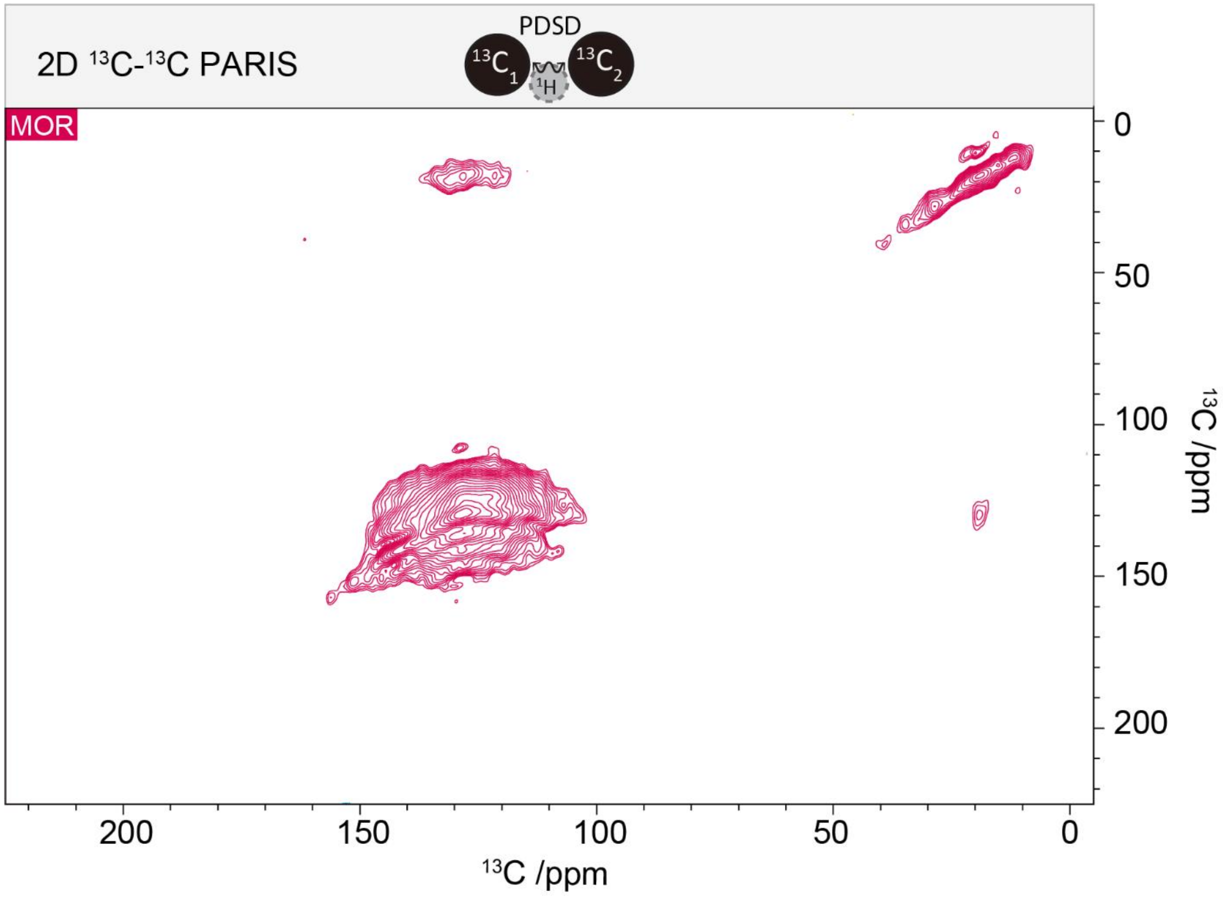

Supplementary Fig. 11 2D ^{13}C-^{13}C DE-based proton-driven spin-diffusion (PDSD), using phase-alternated recoupling irradiation schemes (PARIS), MAS ssNMR correlation spectra of zeolite MOR trapped species to probe all molecules. Herein, ^{13}C atoms were polarized by DE, and a 200 ms PARIS mixing period was used (MAS: magic angle spinning (16KHz), DE: direct excitation). Spectra of trapped products obtained on respective post-reacted zeolites after the hydrogenation of fully isotope-enriched $^{13}CO_2$ in the reactant feed ($^{13}CO_2$ at 30 bar, 375ºC, H_2/$^{13}CO_2$=3, and 10000 mL·g^{-1}·h^{-1} at a time-on-stream of 48 hours).

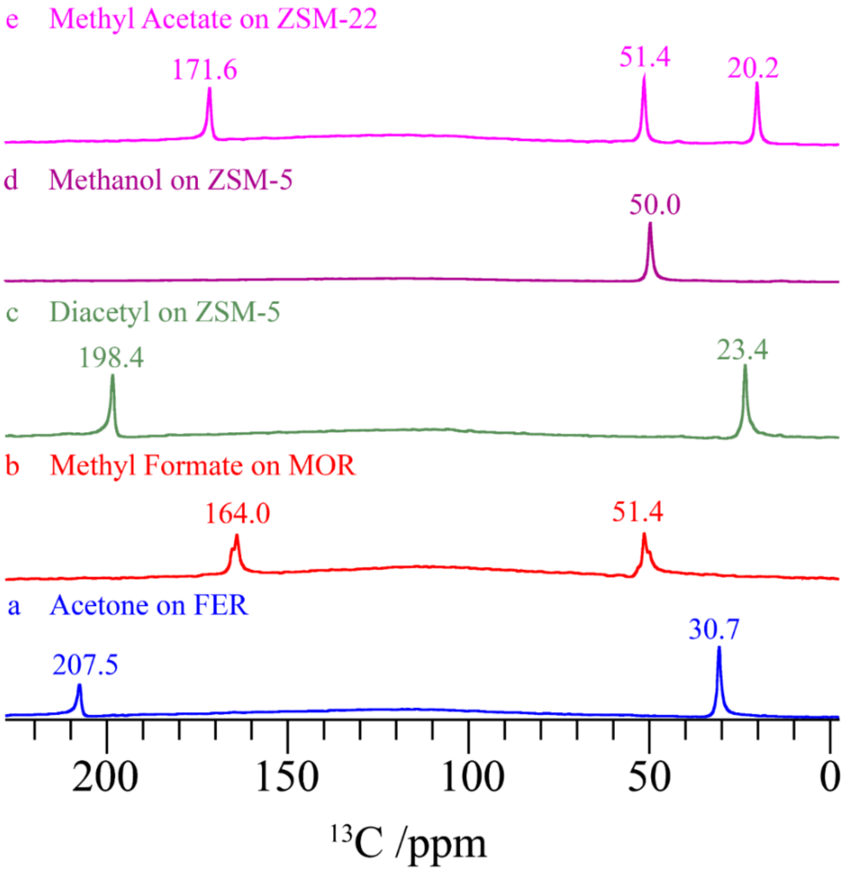

Supplementary Fig. 12 1D ^{3}C MAS ssNMR spectra of organic compounds chemisorbed on zeolites: (a) acetone on zeolite FER, (b) methyl formate on zeolite MOR, (c) diacetyl on zeolite ZSM-5, (d) methanol on zeolite ZSM-5, (e) methyl acetate on zeolite ZSM-22 (Magic Angle Spinning (MAS):16KHz). For the sake of comparison, following responses were observed on post-reacted zeolites after the reaction in Fig. 4e,5f,6c: (i) acetone on FER: 211.9 ppm ($CH_3\underline{C}OCH_3$)/29.8 ppm ($\underline{C}H_3CO\underline{C}H_3$), (ii) diacetyl on ZSM-22: 198.9 ppm ($(CH_3\underline{C}O)_2$)/24.1 ppm ($(\underline{C}H_3CO)_2$), (iii) diacetyl on ZSM-5: 197.5 ppm ($(CH_3\underline{C}O)_2$)/23.0 ppm ($(\underline{C}H_3CO)_2$), (iv) methanol on ZSM-5: 52.1 ppm ($\underline{C}H_3OH$), (v) surface acetate on ZSM-22: 180.2 ppm ($CH_3\underline{C}O$-zeolite)/18.7 ppm ($\underline{C}H_3CO$-zeolite), (vi) surface acetate on FER: 176.6 ppm ($CH_3\underline{C}O$-zeolite)/19.2 ppm ($\underline{C}H_3CO$-zeolite), and (vii) methyl acetate on FER: 177.5 ppm ($CH_3\underline{C}OOCH_3$)/50.7 ppm ($CH_3COO\underline{C}H_3$)/18.4 ppm ($\underline{C}H_3COOCH_3$).

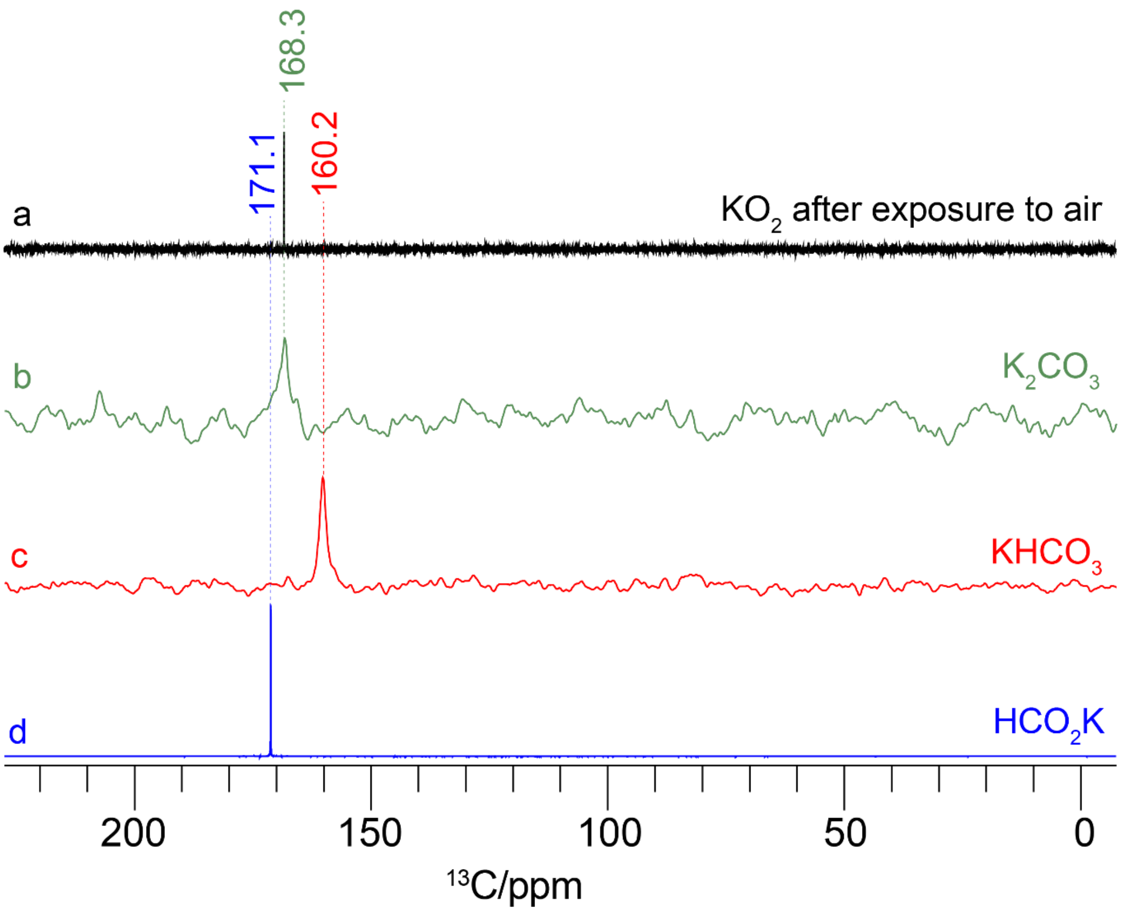

Supplementary Fig. 13 1D ^{13}C DE NMR spectra of potassium salts: (a) air-exposed KO_2 (liquid NMR in D_2O), (b) K_2CO_3 (MAS ssNMR), (c) $KHCO_3$ (MAS ssNMR), (d) HCO2K (liquid NMR in D_2O). The identical peak position among air-exposed KO_2 and K_2CO_3 clearly reveals that KO_2 alone is enough to do direct CO_2 capture from air. Herein, the required amount of KO_2 was transferred to a glass vial for the air exposure over the night, when the solid turned into a liquid as a result of the capture of both water (moisture) and CO_2 from the air. Moreover, neither of these resonances matched the organic carbonyls observed after the hydrogenation of CO_2 reaction. Hence, the observed carbonylated peak on the post-reacted zeolite materials in this study are purely organic in nature (MAS: magic angle spinning (16KHz), DE: direct excitation).

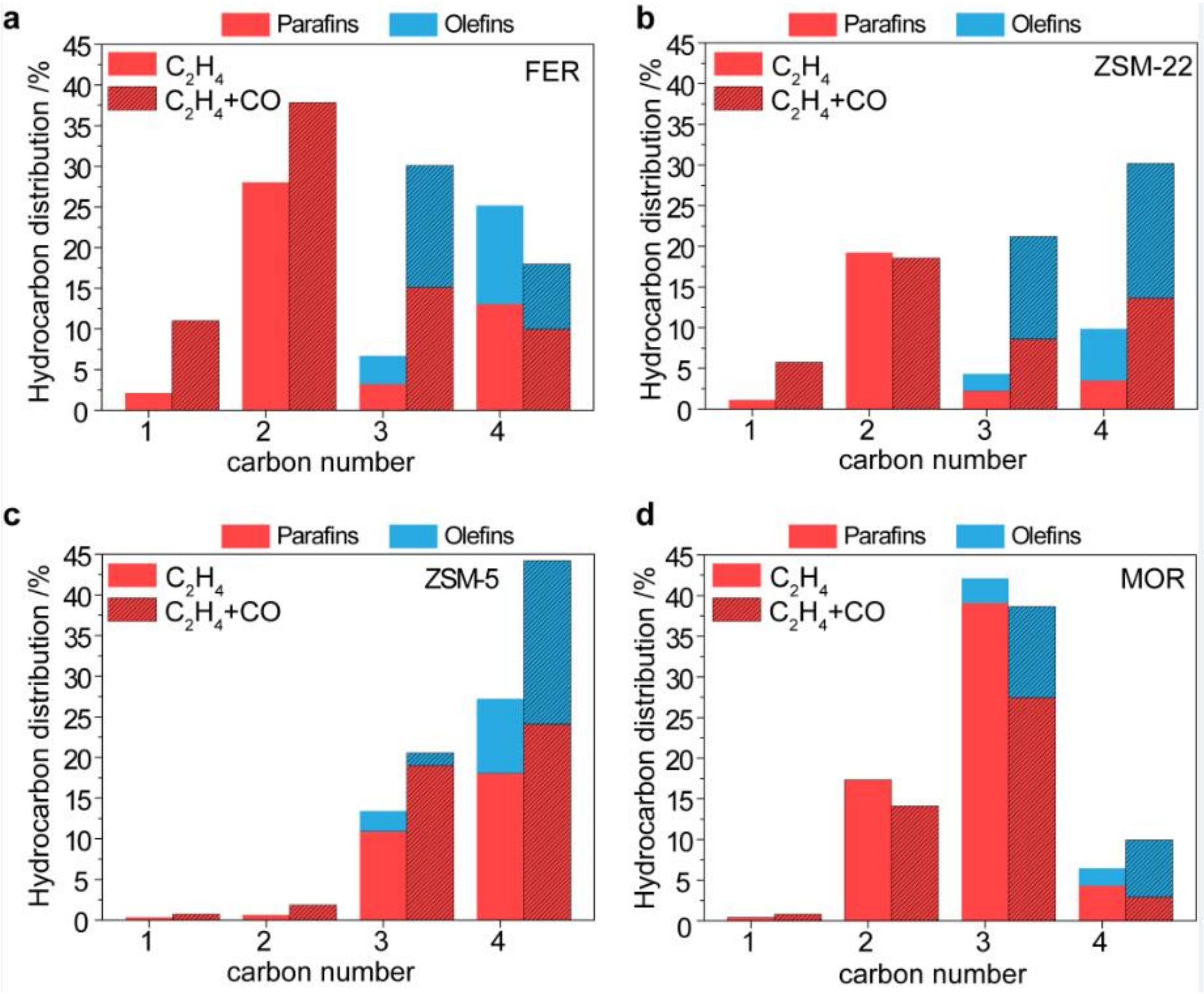

Supplementary Fig. 14 The short-range (C_1-C_4) hydrocarbon distributions for selected standalone zeolites during the control experiments: a, FER. b, ZSM-22. c, ZSM-5, and d, MOR (C_1-C_4-paraffins in red and C_1-C_4-olefins in blue). This set of control experiments clearly demonstrate the effect of incorporating CO into zeolite to initiate the formation of shorter olefins. Hence, these zeolites alone can consume CO to promote desired products and control/alter the final product selectivity. These control experiments were performed on these stand-alone zeolites individually using (i) only (^{12}C)ethylene or (ii) both ^{13}CO and (^{12}C)ethylene in the reactant feed at 30 bar, 375ºC, and 10000 mL·g^{-1}·h^{-1} under hydrogen-rich environment at a time-on-stream of 2 hours (i.e., without any Fe_2O_3@KO_2 phase).

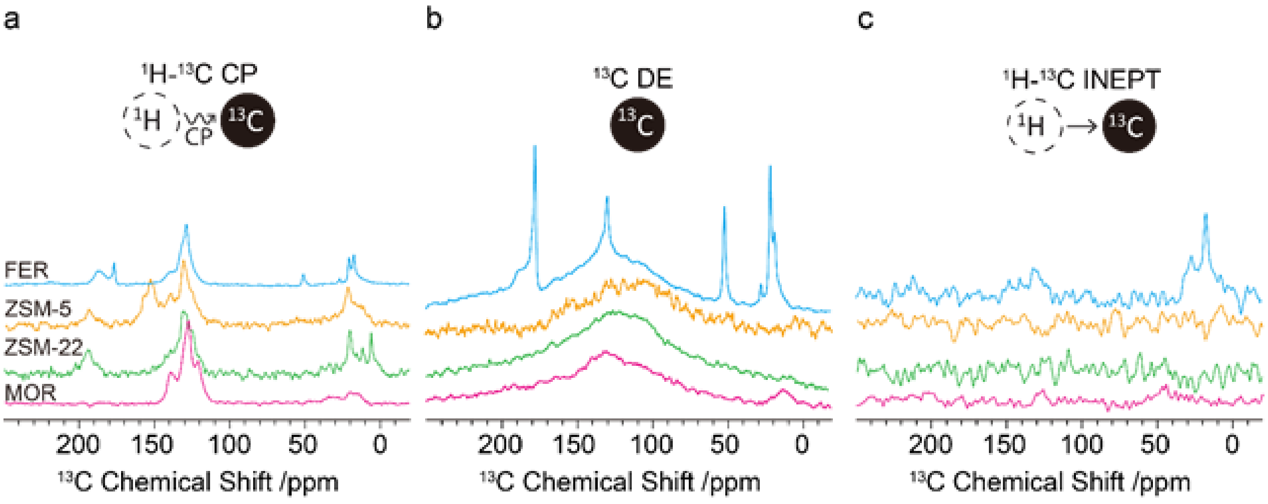

Supplementary Fig. 15 1D ^{3}C MAS ssNMR spectra of post-reacted four zeolites trapped products after the control experiments: (a) ^{1}H-^{13}C CP, (b) ^{13}C DE (same as Fig. 6c), and (c) ^{1}H-^{13}C INEPT (16 KHz magic angle spinning (MAS)). In general, 1D ssNMR spectroscopy (CP, DE, and INEPT) on these post-reacted zeolites revealed the presence of similar organic functional groups: (i) aliphatic/methyl, (ii) olefinic/aromatic, and (iii) carbonyl groups. Hence, the resemblance of catalysis and ssNMR spectroscopic features between usual reaction (Fig. 6b vs. Fig. 2a) and control reactions involving ^{13}CO and (^{12}C) ethylene (Supplementary Fig. 14 and Supplementary Fig.3) is evidently clear. Spectra of trapped products obtained on respective post-reacted zeolites after the control reaction using fully enriched ^{13}CO and (^{12}C) ethylene at 30 bar, 375ºC, and 10000 mL·g^{-1}·h^{-1} under hydrogen-rich environment at a time-on-stream of 2 hours (i.e., without any Fe_2O_3@KO_2 phase).

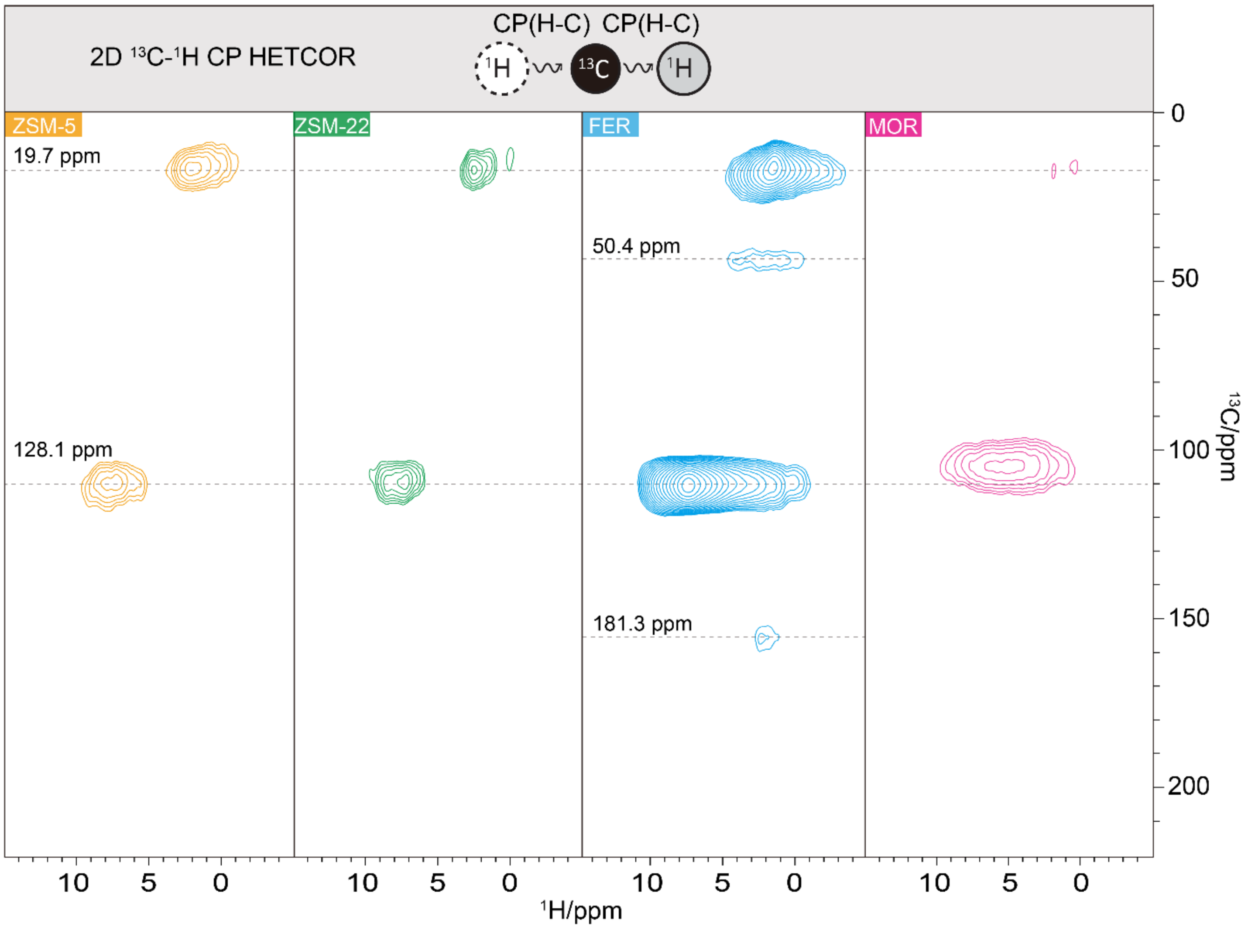

Supplementary Fig. 16 2D ^{13}C-^{1}H CP-based MAS ssNMR correlation spectra of post-reacted four zeolites trapped species to probe rigid molecules after the control experiments (MAS: magic angle spinning (20KHz), CP: cross-polarization, ^{1}H-^{3}C CP contact time: 500µs, p16: ^{13}C-^{1}H CP contact time: 500µs). These CP-based ^{13}C-^{1}H experiments (expectedly) demonstrated the presence of alkylated olefins/aromatics and paraffins mostly. Spectra of trapped products obtained on respective post-reacted zeolites after the control reaction using fully enriched ^{13}CO and (^{12}C) ethylene at 30 bar, 375°C, and 10000 mL·g^{-1}·h^{-1} under hydrogen-rich environment at a time-on-stream of 2 hours (i.e., without any Fe_2O_3@KO_2 phase).

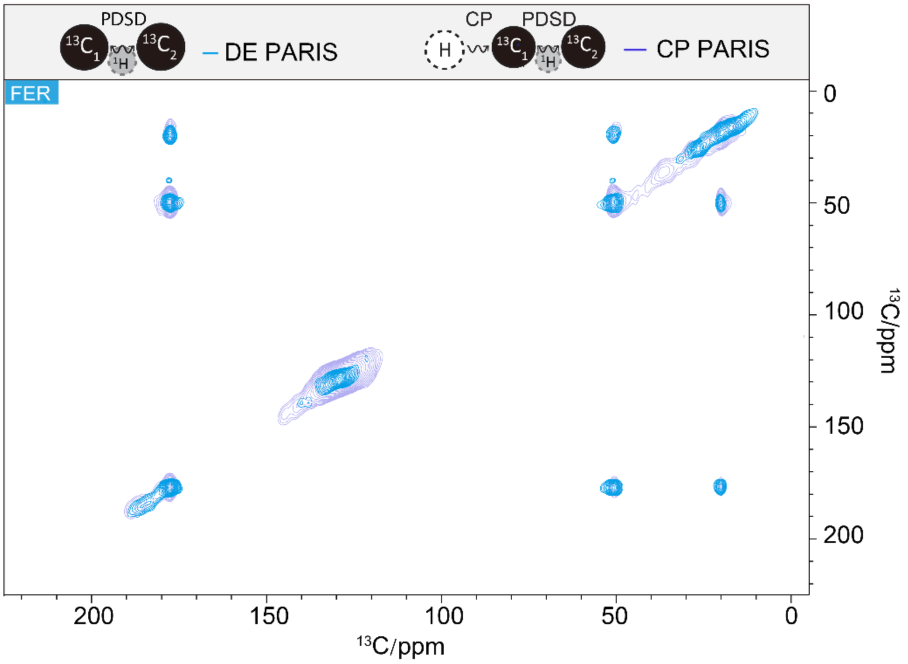

Supplementary Fig. 17 2D ^{13}C-^{13}C DE and CP-based proton-driven spin-diffusion (PDSD), using phase-alternated recoupling irradiation schemes (PARIS), MAS ssNMR correlation spectra of zeolite FER trapped species to probe all molecules after the control experiments. Herein, ^{13}C atoms were polarized by DE (in deep blue) or CP (light blue), and a 200 ms PARIS mixing period was used (MAS: magic angle spinning (16KHz), DE: direct excitation, CP: Cross-polarization). Spectra of trapped products obtained on respective post-reacted zeolites after the control reaction using fully enriched ^{13}CO and (^{12}C)ethylene at 30 bar, 375ºC, and 10000 mL·g^{-1}·h^{-1} under hydrogen-rich environment at a time-on-stream of 2 hours (i.e., without any Fe_2O_3@KO_2 phase).

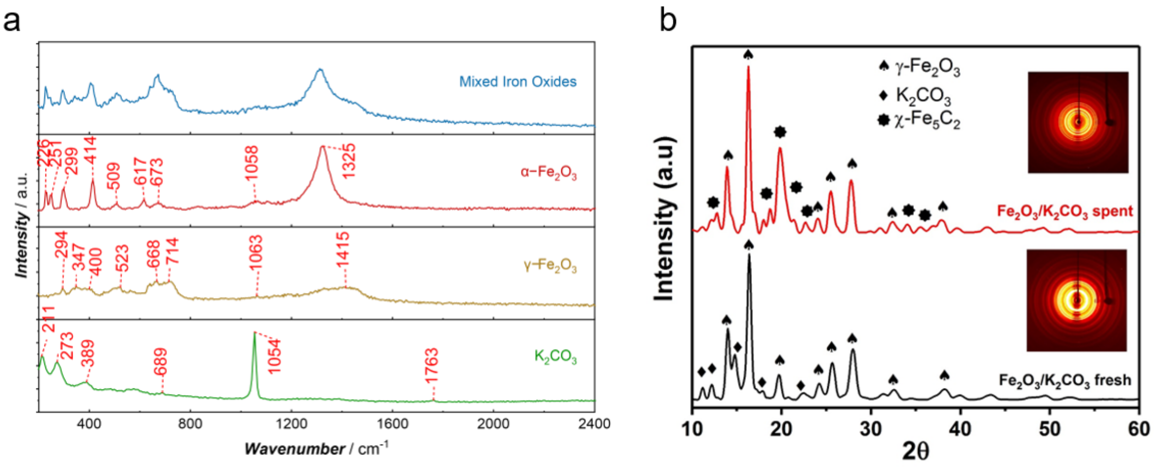

Supplementary Fig. 18 Characterization of Fe/K-metallic phase of the catalyst: (a) Raman spectra (532 nm laser-line) of pristine $Fe_2O_3@K_2CO_3$ catalyst (4 different spots on the surface) and (b) air-sealed capillary single crystal X-Ray diffraction patterns of the fresh and spent $Fe_2O_3@K_2CO_3$ catalyst. (a) Typical α-Fe_2O_3 Raman shifts (226, 251, 299, 414, 509, 617, 673, and 1325 cm^{-1})[73–75] and γ-Fe_2O_3 Raman shifts (294, 347, 400, 523, 668, 714, 1415 cm^{-1})[73,75,76] were observed. Besides; we detected K_2CO_3 related vibrations (211, 273, 389, 689, 1054, 1763 cm^{-1})[77,78] too. (b) The mixed γ-Fe_2O_3/ K_2CO_3-phase was identified on the fresh catalyst, while, in addition, χ-Fe_5C_2 (i.e., the active FTS-phase) has been further confirmed by air sealed capillary XRD on the spent catalysts. During the Raman experiment, the laser-induced heating mediated transformation from maghemite to hematite can be suggested[28–30]. Nevertheless, no organic carbonylated-type of reaction intermediates was detected; only numerous forms of K and Fe-phases were identified.

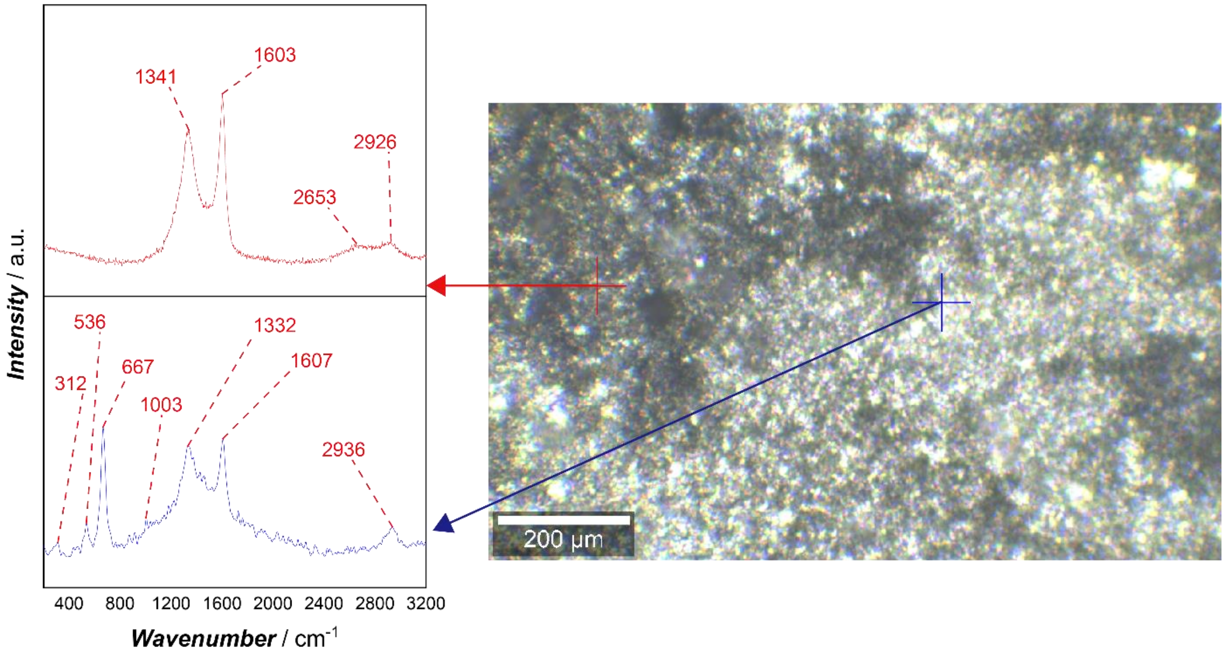

Supplementary Fig. 19 Raman spectra (532 nm laser-line) on the spent Fe_2O_3@K_2CO_3 catalyst: While Raman vibrations raised from carbon species are dominating the spectrum; Fe_3O_4 related signals (i.e., 312, 536, 667 cm^{-1})[30,73,75] were also detectable at some spots (i.e., blue cross).

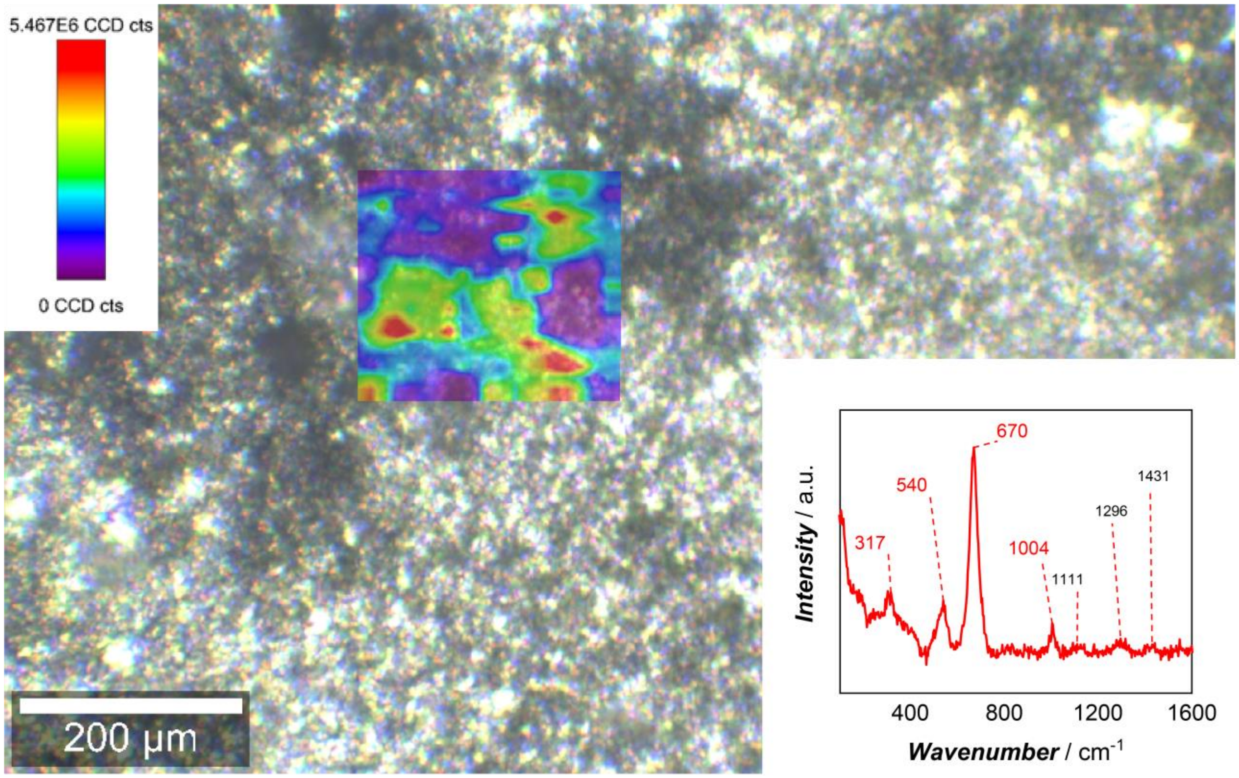

Supplementary Fig. 20 Raman microscopy mapping (633 nm laser-line) for inorganic parts on the spent Fe_2O_3@K_2CO_3 catalyst: The mapping reveals the heterogeneous nature of the catalyst during the reaction. Besides the Raman shifts (i.e., 317, 540, 670 cm^{-1}) indicating the presence of Fe_3O_4; the vibration modes above 1000 cm^{-1} are most likely related to K containing carbonyl groups (i.e., $KHCO_3$, KOOCH) we identified before[2].

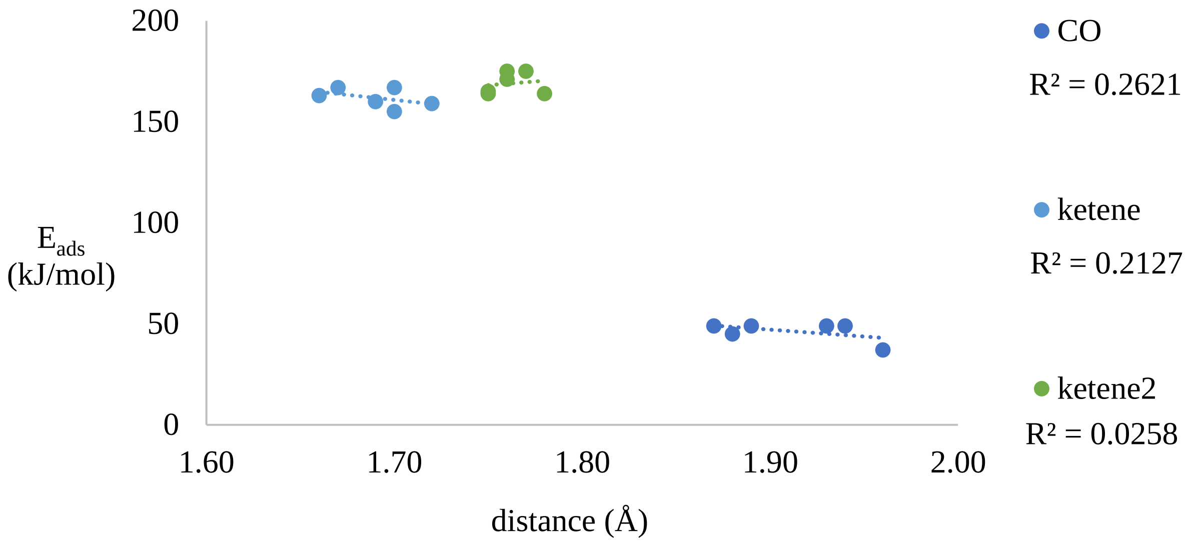

Supplementary Fig. 21 Energetic and geometric analysis. Plot of O-H distance between the zeolite acid site and main reactants and the adsorption energies of CO (dark blue), ketene adsorbed trough O (light blue) and ketene adsorbed trough C (green) presented in Ångstroms and kJ/mol.

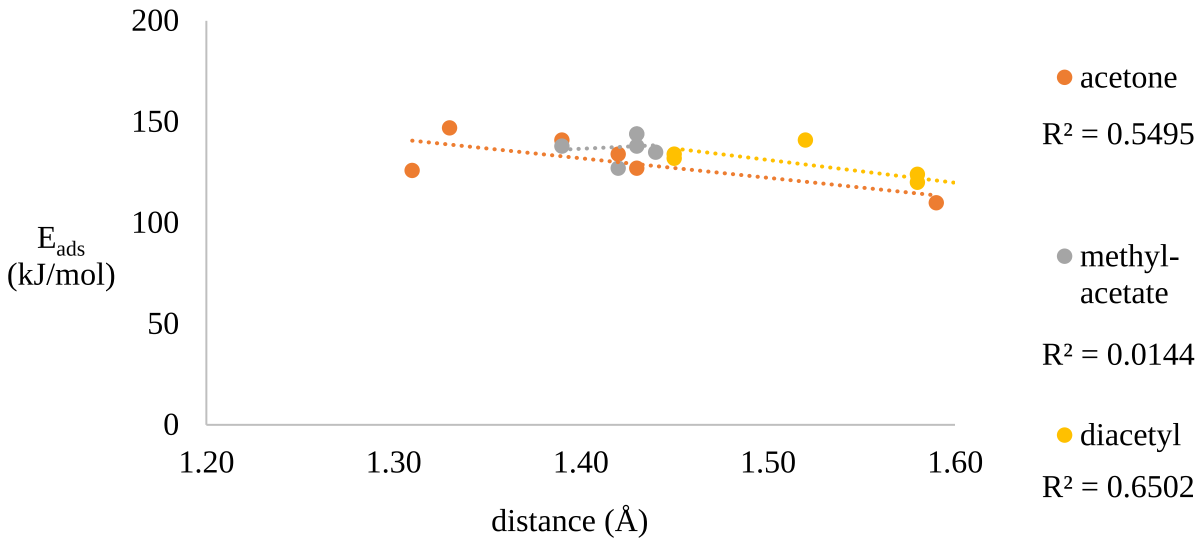

Supplementary Fig. 22 Energetic and geometric analysis. Plot of O-H distance between the zeolite acid site and intermediates and the adsorption energies of acetone (orange), methyl-acetate (grey), diacetyl (yellow), presented in Ångstroms and kJ/mol.

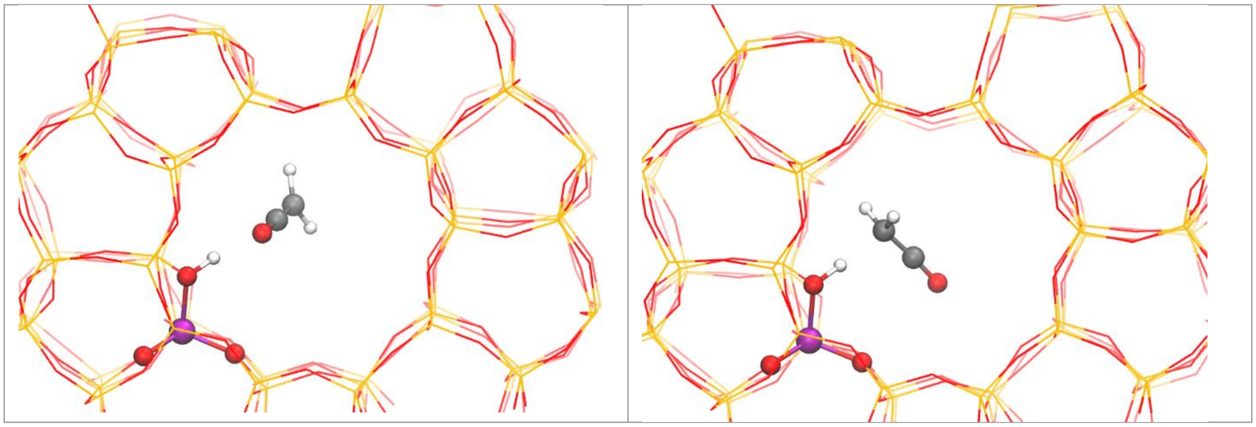

Supplementary Fig. 23 Example of ketene adsorption via O (left) and via C (right) of the H-ZSM-5 [T12] geometrically optimized models, with H – white, O – red, C– grey, Si – yellow, Al – purple.

a

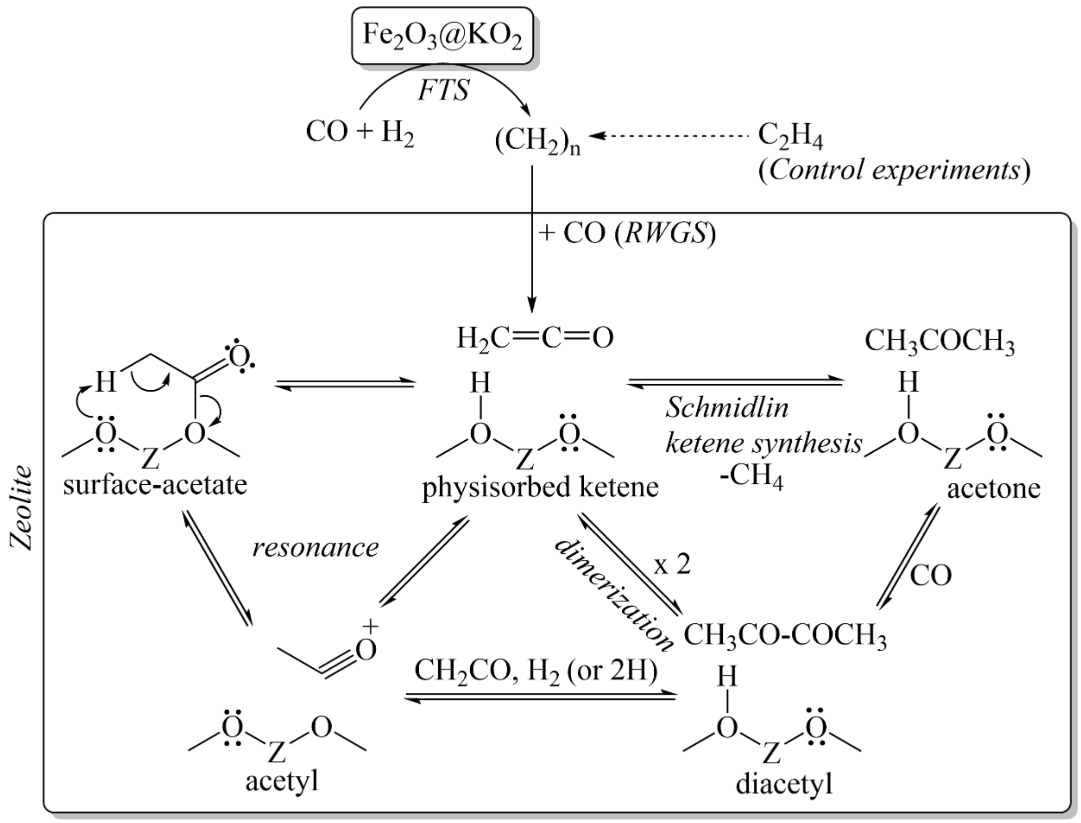

b

[i] $(CH_3CO)_2 \rightleftharpoons CH_3CO + CH_3CO$

[ii] $(CH_3CO)_2 \rightleftharpoons CH_3 + CO + CH_2CO$

[iii] $CH_2CO + H \rightleftharpoons CH_3CO$

[iv] $CH_2CO \rightleftharpoons CH_2 + CO$

[v] $CH_2CO + CH_2 \rightleftharpoons C_2H_4 + CO$

[vi] $(CH_3CO)_2 + H \rightleftharpoons CH_2CO + CH_3CO + H_2$

[vii] $CH_2CO + CH_2 \rightleftharpoons C_2H_5 + CO$

[viii] $(CH_3CO)_2 + CH_3 \rightleftharpoons CH_3COCH_3 + CH_3CO$

[ix] $(CH_3CO)_2 + CH_3 \rightleftharpoons CH_2COCOCH_3 + CH_4$

[x] $CH_2COCOCH_3 \rightleftharpoons CH_2CO + CH_3CO$

[xi] $(CH_3CO)_2 + CH_3 \rightleftharpoons CH_2CO + CH_3CO + CH_4 \rightleftharpoons CH_3COCH_3 + CH_3CO$

Supplementary Fig. 24 Simplified illustration of ketene's plausible mechanistic involvement as a reaction intermediate: a, the proposed interconversion between ketene and other carbonylated intermediates (surface acetate, acetone, diacetyl) on zeolite. **b,** the established combustion chemistry of ketene from literature[53–56,58]. (RWGS: Reverse Water Gas Shift Reaction, FTS: Fischer-Tropsch Synthesis, Z: Zeolites).

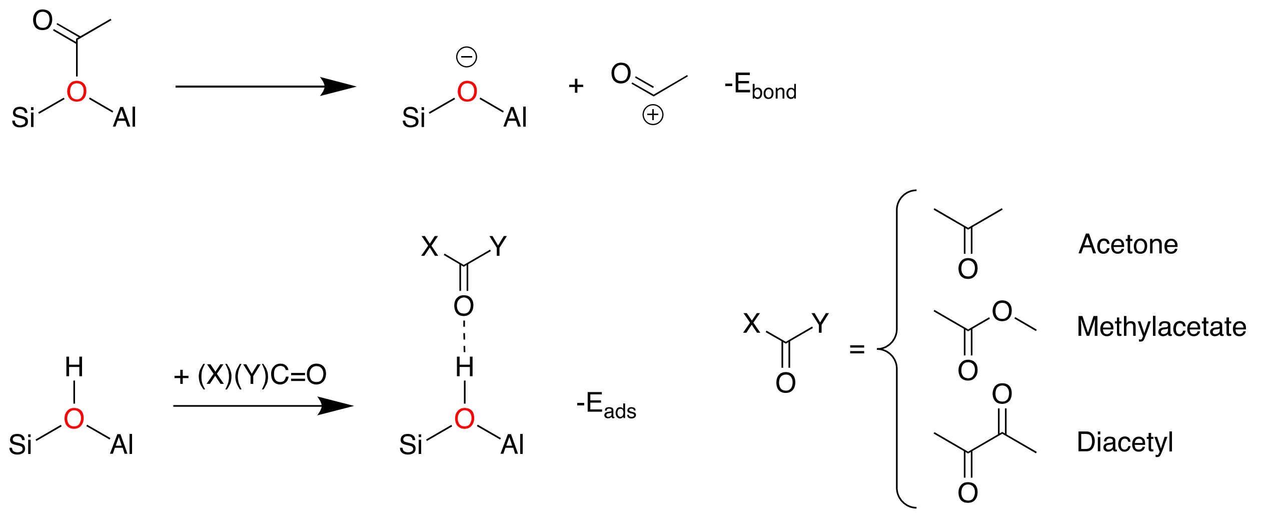

Supplementary Fig. 25 Processes investigated by DFT methods. Specifically, diacetyl conversion to ketene (top row) and carbonylates adsorption (bottom row).

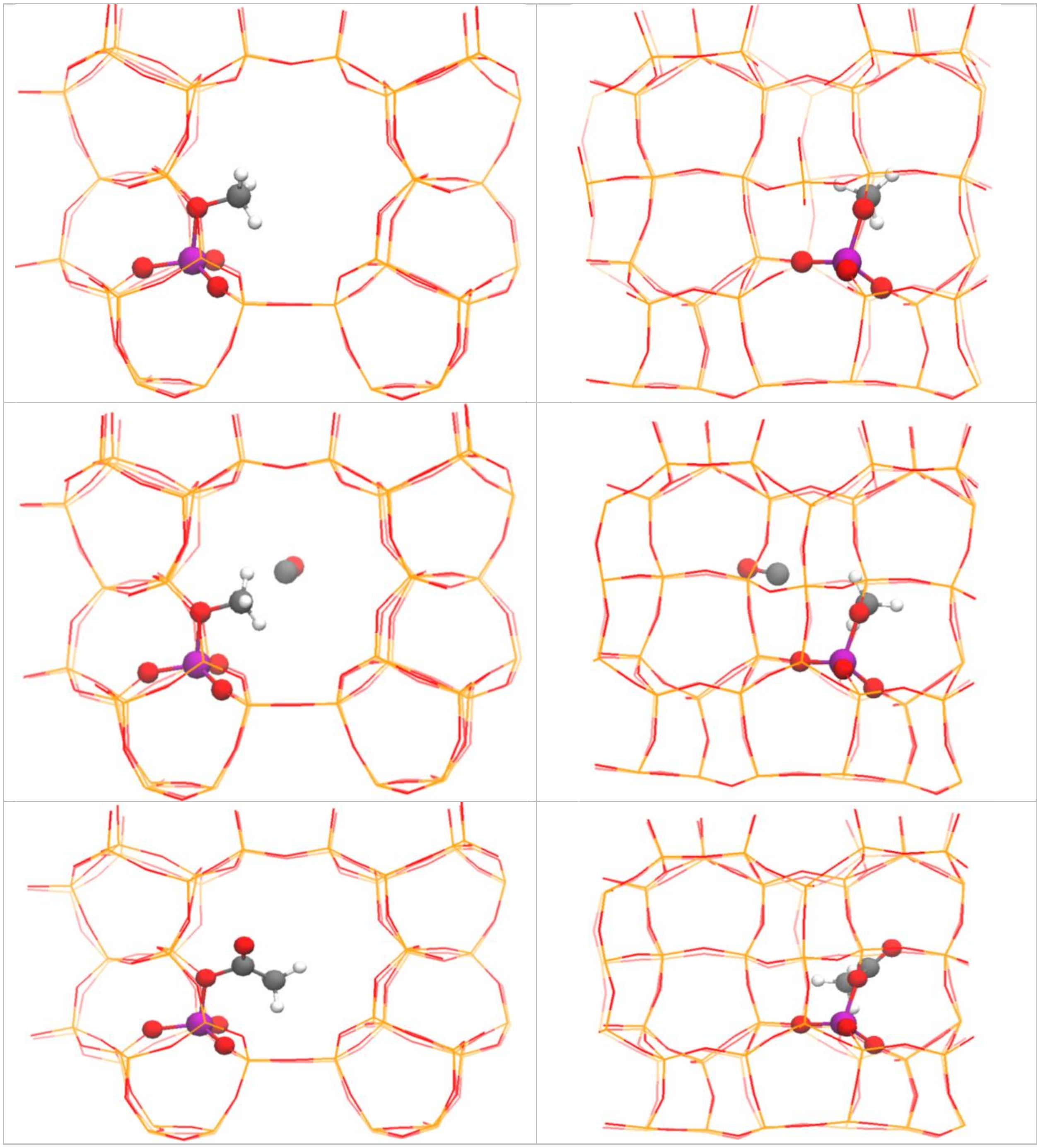

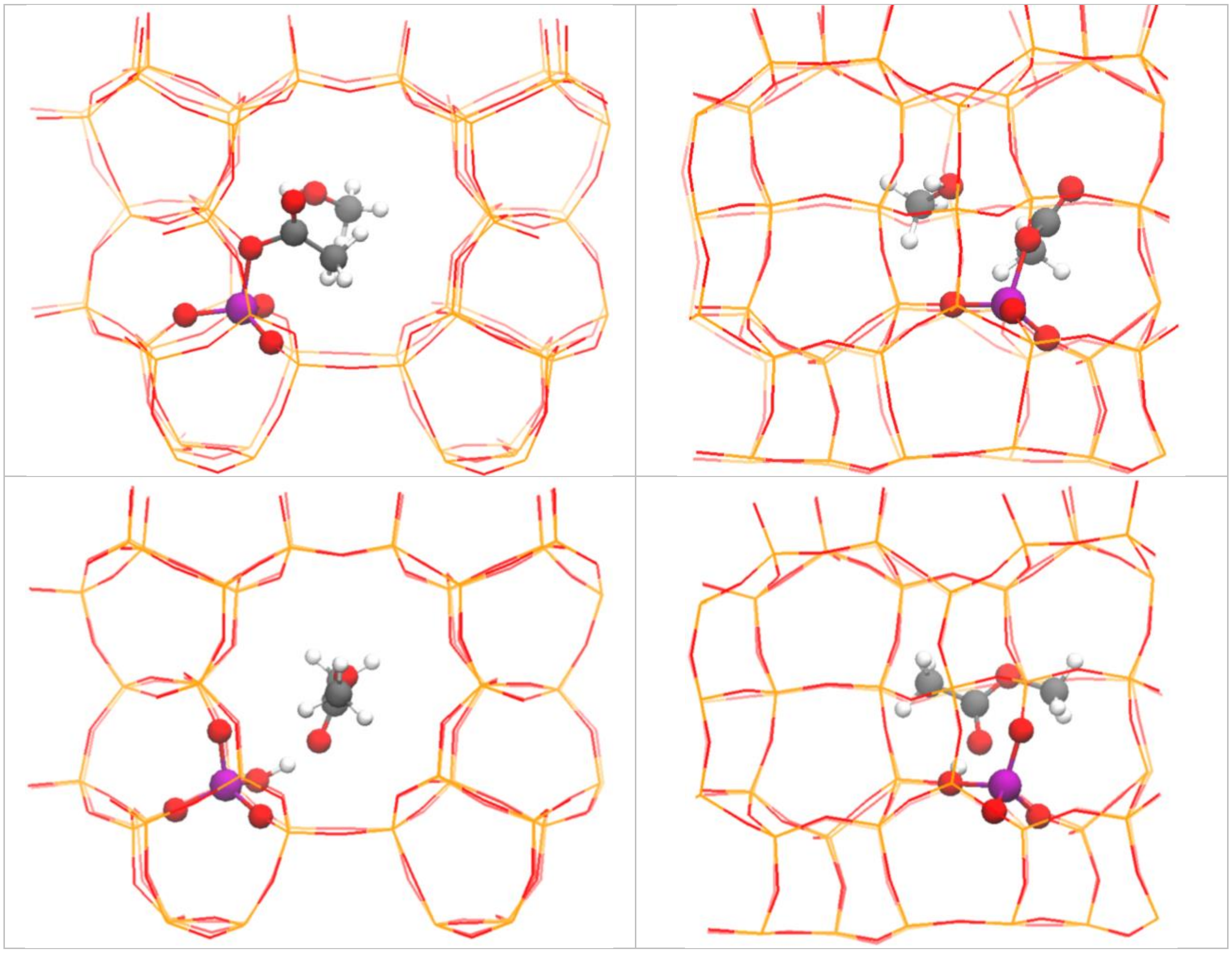

Supplementary Fig. 26 Illustration of reaction route to methyl acetate via surface acetate, with atom colours as follows: Al – purple, Si – yellow, O – red, C – grey, H – white.

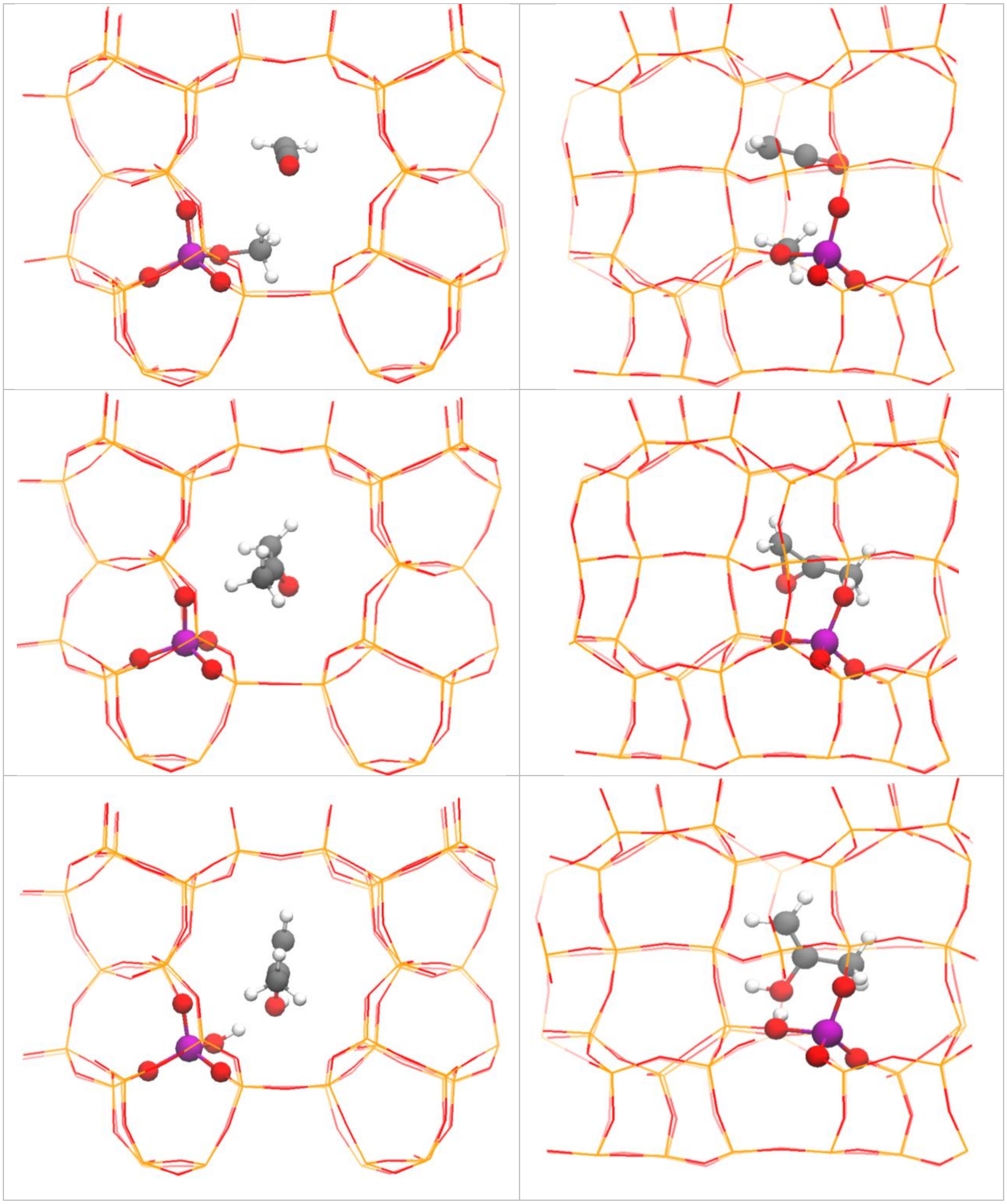

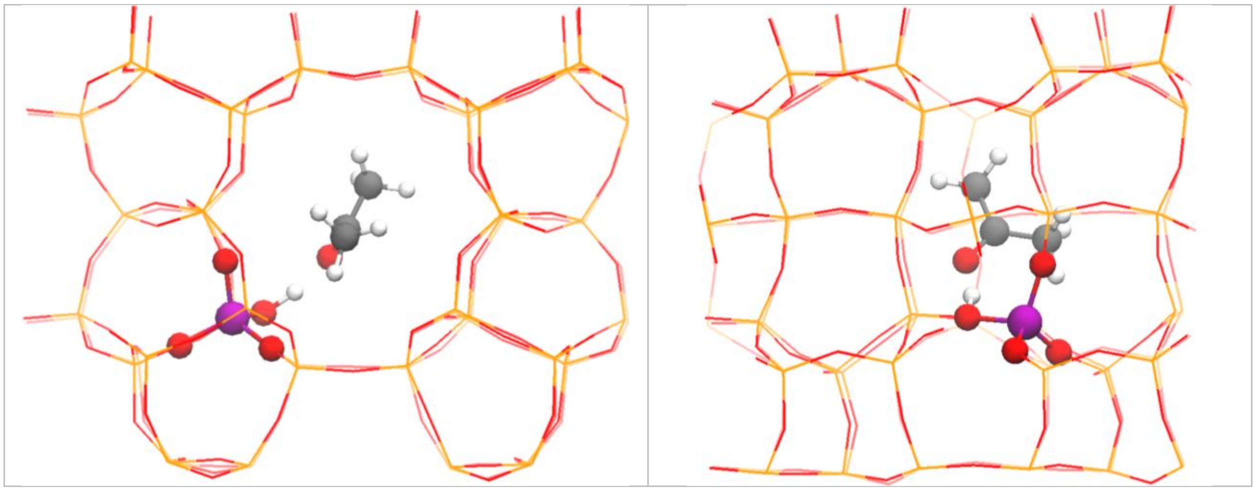

Supplementary Fig. 27 Illustration of reaction route to acetone via ketene methylation and keto-enol tautomerisation, with atom colours as follows: Al – purple, Si – yellow, O – red, C – grey, H – white.

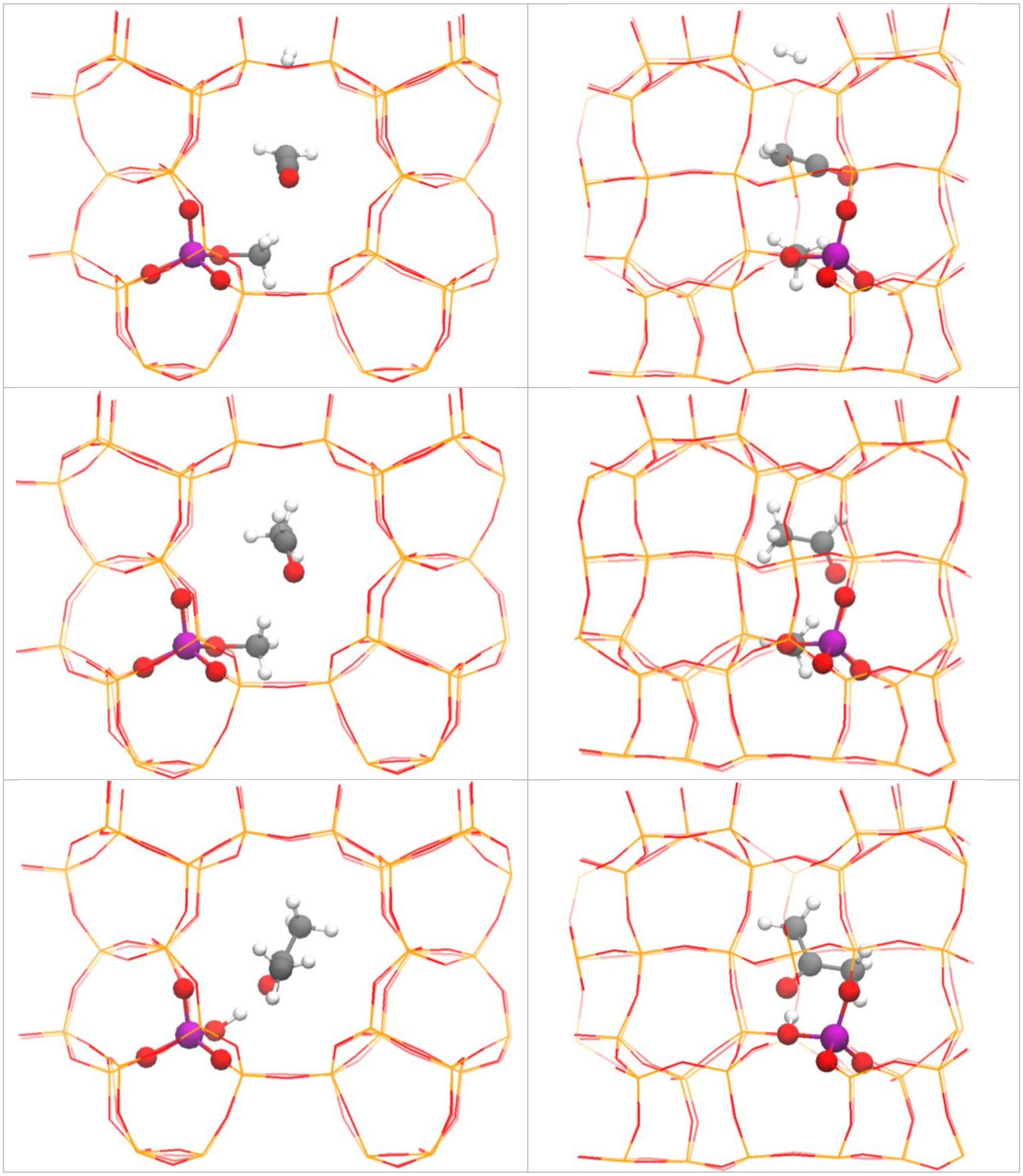

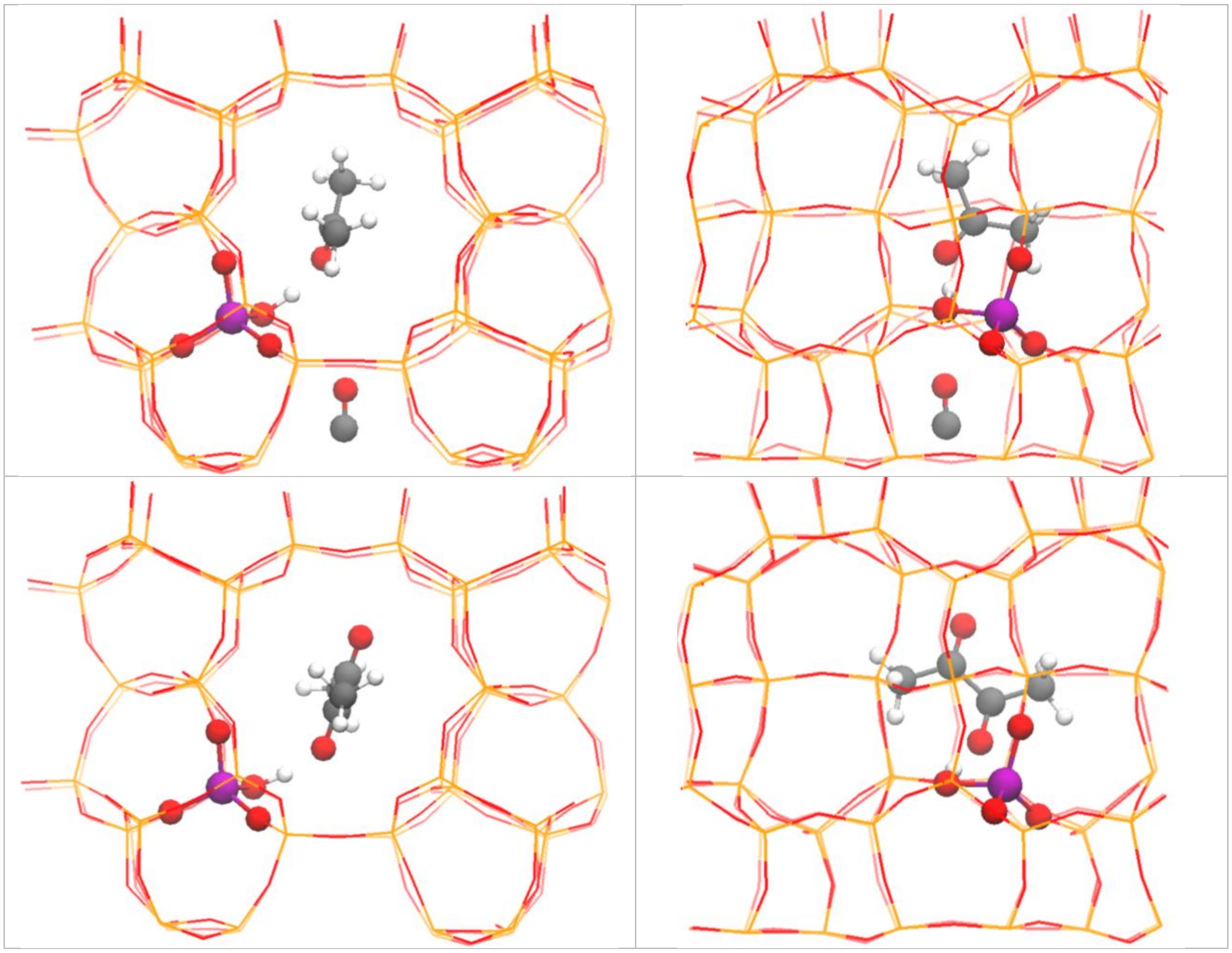

Supplementary Fig. 28 Illustration of reaction route to acetone and diacetyl via ketene hydrogenation acetaldehyde methylation, with atom colours as follows: Al – purple, Si – yellow, O – red, C – grey, H – white.

Supplementary Tables

Supplementary Table 1 Structural details of all zeolites.

**Zeolite**	**Topology**	**Channel Dimensionality**	**Largest member ring**	**SiO_2/Al_2O_3**
ZSM-5	MFI	3D	10	26
Mordenite	MOR	2D	12	20
SAPO-34	CHA	3D	8	0.5
ZSM-22	TON	1D	10	65
Ferrierite	FER	2D	10	20
BETA	BEA	3D	12	38
ZSM-58	DDR	2D	8	25
Y	FAU	3D	12	30

Supplementary Table 2 Structural and textural properties of all zeolites.

**Zeolite (Structure)**	**Silica/ Alumina**	**S_{BET} (m^2/g)**	**$S_{meso/ext}$ (m^2/g)†**	**S_{micro} (m_2/g)‡**	**V_{total} (ml/g)$^{\Delta}$**	**V_{micro} (ml/g) †**	**NH_3 desorb/mass (umol/g)**
ZSM-5 (3D/10MR)	26	417	121	296	0.23	0.12	566.9
BETA (3D/12MR)	38	656	63	493	0.33	0.20	272.5
FER (2D/10MR)	20	397	42	355	0.18	0.13	455.8
MOR (2D/12MR)	20	551	62	489	0.24	0.19	543.6
SAPO-34 (3D/8MR)	0.5	770	8	762	0.28	0.27	666.3
Y (3D/12MR)	30	924	238	686	0.50	0.27	293.1
ZSM-22 (1D/10MR)	65	67	42	25	0.10	0.01	183.3
ZSM-58 (2D/8MR)	25	386	45	341	0.16	0.13	195.4

† $S_{meso/ext} = S_{BET} – S_{micro}$. ‡ From N_2 adsorption isotherm using the t-plot method. $^{\Delta}$ Single point adsorption total pore volume @ p/po=0.95

Supplementary Table 3 Energetic and geometric analysis. Summary of statistical analysis, with lowest (Min) and highest (Max), range and standard deviation of the thermodynamic observables in Table 1 of the main manuscript, presented in kJ/mol.

	Min	Max	Range	Ave	Std.Dev
**CO**	37	49	12	46	5
**ketene (O)**	155	167	12	162	5
**ketene (C)**	164	175	11	169	5
**methyl-acetate**	127	144	17	138	6
**diacetyl**	111	141	30	127	11
**acetone**	110	147	37	131	13
**Zeo-acetate bond**	919	995	76	951	30
**ketene···H$^+$**	37	49	12	46	5

Supplementary Table 4 A brief summary of solid-state NMR assignments. Data from both rigid (denoted as R, primarily from CP-based experiments) and mobile (denoted as M, primarily from DE and INEPT-based experiments) molecules/fragments, observed in this work.

		ZSM-5	ZSM-22	MOR	FER
M1	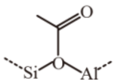		√		√
M2	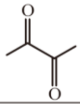	√	√		
M3	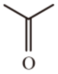	√			√
M4		√	√		
M5	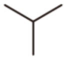	√			
M6	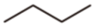			√	√
M7	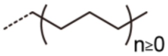				√
M8	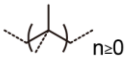	√	√		
R1	CH_3OH	√			
R2	CH_3OCH_3	√			
R3	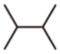		√		
R4	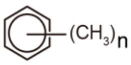	√	√	√	
R4	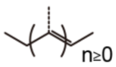			√	

Supplementary References

1. Kumita, Y., Gascon, J., Stavitski, E., Moulijn, J. A. & Kapteijn, F. Shape selective methanol to olefins over highly thermostable DDR catalysts. *Appl. Catal. A Gen.* **391**, 234–243 (2011).
2. Ramirez, A. *et al.* Tandem conversion of CO2 to valuable hydrocarbons in highly concentrated potassium iron catalysts. *ChemCatChem* **11**, 2879–2886 (2019).
3. Ramirez, A. *et al.* Coated sulfated zirconia/SAPO-34 for the direct conversion of CO2 to light olefins. *Catal. Sci. Technol.* **10**, 1507–1517 (2020).
4. Ramirez, A. *et al.* Effect of zeolite topology and reactor configuration on the direct conversion of CO2 to light olefins and aromatics. *ACS Catal.* **9**, 6320–6334 (2019).
5. Dokania, A. *et al.* Acidity modification of ZSM-5 for enhanced production of light olefins from CO2. *J. Catal.* **381**, 347–354 (2020).
6. Chowdhury, A. D. *et al.* Initial carbon-carbon bond formation during the early stages of the methanol-to-olefin process proven by zeolite-trapped acetate and methyl acetate. *Angew. Chem. Int. Ed.* **55**, 15840–15845 (2016).
7. Plessow, P. N. & Studt, F. Unraveling the mechanism of the initiation reaction of the methanol to olefins process using ab Initio and DFT calculations. *ACS Catal.* **7**, 7987–7994 (2017).
8. Liu, Y. *et al.* Formation Mechanism of the First Carbon-Carbon Bond and the First Olefin in the Methanol Conversion into Hydrocarbons. *Angew. Chemie Int. Ed.* **55**, 5723–5726 (2016).
9. Kresse, G. & Furthmüller, J. Efficiency of ab-initio total energy calculations for metals

and semiconductors using a plane-wave basis set. *Comput. Mater. Sci.* **6**, 15–50 (1996).

10. Kresse, G. & Hafner, J. Ab initio molecular-dynamics simulation of the liquid-metalamorphous- semiconductor transition in germanium. *Phys. Rev. B* **49**, 14251–14269 (1994).

11. Perdew, J. P., Burke, K. & Ernzerhof, M. Generalized gradient approximation made simple. *Phys. Rev. Lett.* **77**, 3865–3868 (1996).

12. Grimme, S., Antony, J., Ehrlich, S. & Krieg, H. A consistent and accurate ab initio parametrization of density functional dispersion correction (DFT-D) for the 94 elements H-Pu. *J. Chem. Phys.* **132**, 154104 (2010).

13. Joubert, D. From ultrasoft pseudopotentials to the projector augmented-wave method. *Phys. Rev. B - Condens. Matter Mater. Phys.* **59**, 1758–1775 (1999).

14. John, M., Alexopoulos, K., Reyniers, M. F. & Marin, G. B. Effect of zeolite confinement on the conversion of 1-butanol to butene isomers: Mechanistic insights from DFT based microkinetic modelling. *Catal. Sci. Technol.* **7**, 2978–2997 (2017).

15. Ghorbanpour, A., Rimer, J. D. & Grabow, L. C. Periodic, vdW-corrected density functional theory investigation of the effect of Al siting in H-ZSM-5 on chemisorption properties and site-specific acidity. *Catal. Commun.* **52**, 98–102 (2014).

16. Siahrostami, S. *et al.* Exploring Scaling Relations for Chemisorption Energies on Transition-Metal-Exchanged Zeolites ZSM-22 and ZSM-5. *ChemCatChem* **8**, 767–772 (2016).

17. Chu, Y. *et al.* Slight channel difference influences the reaction pathway of methanol-to-olefins conversion over acidic H-ZSM-22 and H-ZSM-12 zeolites. *Catal. Sci. Technol.* **5**, 3507–3517 (2015).

18. Grajciar, L., Areán, C. O., Pulido, A. & Nachtigall, P. Periodic DFT investigation of the effect of aluminium content on the properties of the acid zeolite H-FER. *Phys. Chem. Chem. Phys.* **12**, 1497–1506 (2010).

19. Ramirez, A. *et al.* Effect of Zeolite Topology and Reactor Configuration on the Direct Conversion of CO<inf>2</inf> to Light Olefins and Aromatics. *ACS Catal.* **9**, (2019).

20. Boronat, M. & Corma, A. What is measured when measuring acidity in zeolites with probe molecules? *ACS Catal.* **9**, 1539–1548 (2019).

21. Pines, A., Gibby, M. G. & Waugh, J. S. Proton-enhanced nuclear induction spectroscopy. a method for high resolution nmr of dilute spins in solids. *J. Chem. Phys.* **56**, 1776–1777 (1972).

22. Morris, G. A. & Freeman, R. Enhancement of nuclear magnetic resonance signals by polarization transfer. *J. Am. Chem. Soc.* **101**, 760–762 (1979).

23. Weingarth, M., Demco, D. E., Bodenhausen, G. & Tekely, P. Improved magnetization transfer in solid-state NMR with fast magic angle spinning. *Chem. Phys. Lett.* **469**, 342–348 (2009).

24. Fung, B. M., Khitrin, A. K. & Ermolaev, K. An improved broadband decoupling sequence for liquid crystals and solids. *J. Magn. Reson.* **142**, 97–101 (2000).

25. Bodenhausen, G. & Ruben, D. J. Natural abundance nitrogen-15 NMR by enhanced heteronuclear spectroscopy. *Chem. Phys. Lett.* **69**, 185–189 (1980).

26. Zhou, D. H. & Rienstra, C. M. High-performance solvent suppression for proton detected solid-state NMR. *J. Magn. Reson.* **192**, 167–172 (2008).

27. Weingarth, M., Tekely, P. & Bodenhausen, G. Efficient heteronuclear decoupling by quenching rotary resonance in solid-state NMR. *Chem. Phys. Lett.* **466**, 247–251 (2008).

28. El Mendili, Y., Bardeau, J. F., Randrianantoandro, N., Greneche, J. M. & Grasset, F. Structural behavior of laser-irradiated γ-Fe2O3 nanocrystals dispersed in porous silica matrix : γ-Fe2O3 to α-Fe2O3 phase transition and formation of ε-Fe2O3. *Sci. Technol. Adv. Mater.* **17**, 597–609 (2016).

29. Slavov, L. *et al.* Raman spectroscopy investigation of magnetite nanoparticles in ferrofluids. *J. Magn. Magn. Mater.* **322**, 1904–1911 (2010).

30. Shebanova, O. N. & Lazor, P. Raman study of magnetite (Fe3O4): Laser-induced thermal effects and oxidation. *J. Raman Spectrosc.* **34**, 845–852 (2003).

31. Sole, C., Drewett, N. E. & Hardwick, L. J. Insitu Raman study of lithium-ion intercalation into microcrystalline graphite. *Faraday Discuss.* **172**, 223–237 (2014).

32. Yang, Z., Luo, M., Liu, Q. & Shi, B. In situ XRD and Raman Investigation of the Activation Process over K–Cu–Fe/SiO2 Catalyst for Fischer–Tropsch Synthesis Reaction. *Catal. Letters* **150**, 2437–2445 (2020).

33. Zhang, C. *et al.* Adsorption and reaction of CO and hydrogen on iron-based Fischer-Tropsch synthesis catalysts. *J. Mol. Catal. A Chem.* **328**, 35–43 (2010).

34. Paalanen, P. P., Van Vreeswijk, S. H. & Weckhuysen, B. M. Combined in Situ X-ray Powder Diffractometry/Raman Spectroscopy of Iron Carbide and Carbon Species Evolution in Fe(-Na-S)/α-Al2O3 Catalysts during Fischer-Tropsch Synthesis. *ACS Catal.* 9837–9855 (2020) doi:10.1021/acscatal.0c01851.

35. Warringham, R. *et al.* The application of inelastic neutron scattering to investigate a hydrogen pre-treatment stage of an iron Fischer-Tropsch catalyst. *Appl. Catal. A Gen.* **489**, 209–217 (2015).

36. Pérez, S., Mondragón, F. & Moreno, A. Iron ore as precursor for preparation of highly

active χ-Fe5C2 core-shell catalyst for Fischer-Tropsch synthesis. *Appl. Catal. A Gen.* **587**, 117264 (2019).

37. Yang, C., Zhao, H., Hou, Y. & Ma, D. Fe 5C 2 nanoparticles: A facile bromide-induced synthesis and as an active phase for Fischer-Tropsch synthesis. *J. Am. Chem. Soc.* **134**, 15814–15821 (2012).

38. Ding, M. *et al.* Study on reduction and carburization behaviors of ironbased Fischer-Tropsch synthesis catalyst. *Energy Procedia* **61**, 2267–2270 (2014).

39. Wu, B., Tian, L., Xiang, H., Zhang, Z. & Li, Y. W. Novel precipitated iron Fischer-Tropsch catalysts with Fe3O 4 coexisting with α-Fe2O3. *Catal. Letters* **102**, 211–218 (2005).

40. Tiernan, M. J., Barnes, P. A. & Parkes, G. M. B. Reduction of iron oxide catalysts: The investigation of kinetic parameters using rate perturbation and linear heating thermoanalytical techniques. *J. Phys. Chem. B* **105**, 220–228 (2001).

41. Chernyshova, I. V., Somasundaran, P. & Ponnurangam, S. On the origin of the elusive first intermediate of CO2 electroreduction. *Proc. Natl. Acad. Sci. U. S. A.* **115**, E9261–E9270 (2018).

42. Mitchell, P. C. H., Holroyd, R. P., Poulston, S., Bowker, M. & Parker, S. F. Inelastic neutron scattering of model compounds for surface formates: Potassium formate, copper formate and formic acid. *J. Chem. Soc. - Faraday Trans.* **93**, 2569–2575 (1997).

43. Davis, A. R. & Oliver, B. G. A vibrational-spectroscopic study of the species present in the CO2-H2O system. *J. Solution Chem.* **1**, 329–339 (1972).

44. Jones, A. J. & Iglesia, E. The strength of Brønsted acid sites in microporous aluminosilicates. *ACS Catal.* **5**, 5741–5755 (2015).

45. Niwa, M. & Katada, N. New method for the temperature- programmed desorption (TPD) of ammonia experiment for characterization of zeolite acidity: A review. *Chem. Rec.* **13**, 432–455 (2013).

46. Wang, B. & Manos, G. Role of Strong Zeolitic Acid Sites on Hydrocarbon Reactions. *Ind. Eng. Chem. Res.* **47**, 2948–2955 (2008).

47. Xu, Q. *et al.* Formation and Regeneration of Shape-Selective ZSM-35 Catalysts for n - Butene Skeletal Isomerization to Isobutene. *ACS Omega* **3**, 8202–8211 (2018).

48. Feller, A., Zuazo, I., Guzman, A., Barth, J. O. & Lercher, J. A. Common mechanistic aspects of liquid and solid acid catalyzed alkylation of isobutane with n -butene. *J. Catal.* **216**, 313–323 (2003).

49. Whiting, G. T. *et al.* Multiscale mechanistic insights of shaped catalyst body formulations and their impact on catalytic properties. *ACS Catal.* **9**, 4792–4803 (2019).

50. Wang, C. *et al.* Extra-framework aluminum-assisted initial C−C bond formation in methanol-to-olefins conversion on zeolite H-ZSM-5. *Angew. Chem. Int. Ed.* **57**, 10197–10201 (2018).

51. Fang, H., Zheng, A., Chu, Y. & Deng, F. 13C Chemical shift of adsorbed acetone for measuring the acid strength of solid acids: A theoretical calculation study. *J. Phys. Chem. C* **114**, 12711–12718 (2010).

52. Chowdhury, A. D. *et al.* Bridging the gap between the direct and hydrocarbon pool mechanisms of the methanol-to-hydrocarbons process. *Angew. Chem. Int. Ed.* **57**, 8095–8099 (2018).

53. Christensen, M. & Konnov, A. A. Laminar burning velocity of diacetyl + air flames. Further assessment of combustion chemistry of ketene. *Combust. Flame* **178**, 97–110

(2017).

54. Rice, F. O. & Walters, W. D. The thermal decomposition of diacetyl. *J. Chem. Phys.* **7**, 1015–1018 (1939).

55. Knoll, H., Schliebs, R. & Scherzer, K. On the displacement reaction CH3+CH3 COCOCH3→CH3 COCH3+CH3 CO. *React. Kinet. Catal. Lett.* **8**, 469–475 (1978).

56. Rasmussen, D. B. *et al.* Ketene as a reaction intermediate in the carbonylation of dimethyl ether to methyl acetate over mordenite. *Angew. Chem. Int. Ed.* **54**, 7261–7264 (2015).

57. Chowdhury, A. D. & Gascon, J. The curious case of ketene in zeolite chemistry and catalysis. *Angew. Chem. Int. Ed.* **57**, 14982 –14985 (2018).

58. Allen, A. D. & Tidwell, T. T. Ketenes and other cumulenes as reactive intermediates. *Chem. Rev.* **113**, 7287–7342 (2013).

59. Ross, M. B. *et al.* Designing materials for electrochemical carbon dioxide recycling. *Nat. Catal.* **2**, 648–658 (2019).

60. Jin, L. & Seifitokaldani, A. In situ spectroscopic methods for electrocatalytic CO2 reduction. *Catalysts* **10**, 481 (2020).

61. Handoko, A. D., Wei, F., Jenndy, Yeo, B. S. & Seh, Z. W. Understanding heterogeneous electrocatalytic carbon dioxide reduction through operando techniques. *Nat. Catal.* **1**, 922–934 (2018).

62. Kibria, M. G. *et al.* Electrochemical CO2 reduction into chemical feedstocks: From mechanistic electrocatalysis models to system design. *Adv. Mater.* **31**, 1807166 (2019).

63. Pérez-Gallent, E., Figueiredo, M. C., Calle-Vallejo, F. & Koper, M. T. M. Spectroscopic observation of a hydrogenated CO dimer intermediate during CO reduction on Cu(100) electrodes. *Angew. Chem. Int. Ed.* **56**, 3621 –3624 (2017).

64. Schouten, K. J. P., Qin, Z., Gallent, E. P. & Koper, M. T. M. Two pathways for the formation of ethylene in CO reduction on single-crystal copper electrodes. *J. Am. Chem. Soc.* **134**, 9864–9867 (2012).

65. Kortlever, R., Shen, J., Schouten, K. J. P., Calle-Vallejo, F. & Koper, M. T. M. Catalysts and reaction pathways for the electrochemical reduction of carbon dioxide. *J. Phys. Chem. Lett.* **6**, 4073–4082 (2015).

66. Jiao, F. *et al.* Selective conversion of syngas to light olefins. *Science (80-.).* **351**, 1065–1068 (2016).

67. Çağlayan, M. *et al.* Initial carbon−carbon bond formation during the early stages of methane dehydroaromatization. *Angew. Chem. Int. Ed.* **59**, 16741–16746 (2020).

68. Chowdhury, A. D. *et al.* Electrophilic aromatic substitution over zeolites generates Wheland-type reaction intermediates. *Nat. Catal.* **1**, 23–31 (2018).

69. Chowdhury, A. D. *et al.* Unraveling the homologation reaction sequence of the zeolite-catalyzed ethanol-to-hydrocarbons process. *Angew. Chem. Int. Ed.* **58**, 3908–3912 (2019).

70. Fu, D. *et al.* Elucidating zeolite channel geometry–reaction intermediate relationships for the methanol-to-hydrocarbon process. *Angew. Chem. Int. Ed.* **59**, 20024–20030 (2020).

71. Hemberger, P., Van Bokhoven, J. A., Pérez-Ramírez, J. & Bodi, A. New analytical tools for advanced mechanistic studies in catalysis: Photoionization and photoelectron photoion coincidence spectroscopy. *Catal. Sci. Technol.* **10**, 1975–1990 (2020).

72. Nastase, S. A. F., O'Malley, A. J., Catlow, C. R. A. & Logsdail, A. J. Computational QM/MM investigation of the adsorption of MTH active species in H-Y and H-ZSM-5. *Phys. Chem. Chem. Phys.* **21**, 2639–2650 (2019).

73. Kumar, P., No-Lee, H. & Kumar, R. Synthesis of phase pure iron oxide polymorphs thin

films and their enhanced magnetic properties. *J. Mater. Sci. Mater. Electron.* **25**, 4553–4561 (2014).

74. Shim, S. H. & Duffy, T. S. Raman spectroscopy of Fe2O3 to 62 GPa. *Am. Mineral.* **87**, 318–326 (2002).

75. De Faria, D. L. A., Venâncio Silva, S. & De Oliveira, M. T. Raman microspectroscopy of some iron oxides and oxyhydroxides. *J. Raman Spectrosc.* **28**, 873–878 (1997).

76. Lopez-Sanchez, J. *et al.* Epsilon iron oxide: Origin of the high coercivity stable low Curie temperature magnetic phase found in heated archeological materials. *Geochemistry, Geophys. Geosystems* **18**, 2646–2656 (2017).

77. Berlanga, G. *et al.* Remote Raman spectroscopy of natural rocks. *Appl. Opt.* **58**, 8971 (2019).

78. Buzgar, N. & Ionut Apopei, A. The Raman Study of Carbonates. *Geologie* **55**, 97–112 (2009).
